# Paracetamol and Metformin Reduce NK-Cell Susceptibility in MCF-7 Breast Cancer Cells in Association with Enrichment of Immune-Evasive CD44^+^CD24^−^ Stem-like Subpopulations

**DOI:** 10.3390/ijms27167211

**Published:** 2026-08-12

**Authors:** Nhat Chau Truong, Nhi Thao Huynh, Khanh Gia Trinh, Anh Thuy-Kieu Phan, Duyen Thi-Thuy Le, Phuc Van Pham

**Affiliations:** 1 VNUHCM-US Stem Cell Institute, University of Science, Ho Chi Minh City 70000, Vietnam; nhattruong@sci.edu.vn (N.C.T.);; 2 Vietnam National University Ho Chi Minh City, Ho Chi Minh City 70000, Vietnam

**Keywords:** breast cancer, paracetamol, metformin, natural killer cells, immune evasion, cancer stem cells

## Abstract

Natural Killer (NK) cell-mediated immunosurveillance is a cornerstone of anti-tumor defense, yet its efficacy can be compromised by common clinical medications. Paracetamol (APAP) and metformin (MET) are widely used for pain and metabolic management in cancer patients, but their unintended effects on the immune–tumor interface remain poorly understood. MCF-7 breast cancer cells (Luminal A subtype) were treated with APAP or MET and co-cultured with primary expanded NK cells (CD3^−^CD56^+^CD16^+^). We evaluated cell proliferation, cell cycle distribution, and the enrichment of the CD44^+^CD24^−^ cancer stem-like cell (CSC-like) subpopulation. Transcriptional changes in immune checkpoints (*PD-L1/L2*), stress ligands (*MICA/B*), and costimulatory molecules *CD80/86* were quantified via RT-qPCR. Despite inhibiting MCF-7 growth (IC_50_ at 48 h: 11.86 mM for APAP; 21.11 mM for MET), both drugs induced a “therapeutic paradox” by promoting an immune-evasive phenotype. APAP and MET significantly enriched the CD44^+^CD24^−^ CSC-like subpopulation to 75.31% and 68.31%, respectively, compared to 11.00% in controls. Molecular analysis revealed a robust upregulation of *PD-L1* (19.9-fold by APAP) and *PD-L2* (16.4-fold by MET), alongside increased *CD80/86* and *MICA/B* transcription. Consequently, drug-treated cells exhibited marked resistance to NK-mediated apoptosis and necrosis. NK cells preferentially eliminated non-stem cells (non-CSCs), inadvertently further concentrating the highly resistant CSC-like subpopulation. Additional experiments revealed that APAP directly impaired NK-cell survival and reduced the proportion of CD3^−^CD56^+^ cells, whereas MET exerted minimal effects on NK cells, suggesting distinct mechanisms underlying the observed reduction in NK-mediated cytotoxicity. Under the experimental conditions employed in this study, APAP and MET were associated with reduced susceptibility of MCF-7 cells to NK-mediated killing through distinct but partially overlapping mechanisms, including CSC-like enrichment and transcriptional activation of immune-evasion pathways. Although these findings were obtained in a mechanistic in vitro model using supra-physiological drug concentrations, they identify potential mechanisms that warrant further validation in physiologically relevant experimental systems and in vivo models.

## 1. Introduction

Global health is currently challenged by the rising incidence of breast cancer, which remains the most frequently diagnosed malignancy and the leading cause of cancer-related mortality among women worldwide [1]. In 2022, approximately 2.3 million new cases were diagnosed, resulting in over 666,000 deaths globally [1]. Breast cancer exhibits remarkable heterogeneity, with the Luminal A subtype being the most prevalent, accounting for approximately 60% of all cases. For experimental oncology, the MCF-7 cell line serves as a quintessential model for Luminal A invasive ductal carcinoma, characterized by its ductal epithelial features [2,3].

A critical molecular hallmark of the MCF-7 line is its functional deficiency in caspase-3, resulting from a 47-base pair deletion within exon 3 of the *casp3* gene. This mutation leads to a premature stop codon that completely abrogates the translation of functional caspase-3 protein. As caspase-3 is a central executioner of apoptosis, its absence in MCF-7 cells contributes to their relative resistance to various chemotherapeutic agents [4]. Consequently, anti-tumor immune responses, such as those mediated by Natural Killer (NK) cells, must proceed through caspase-3-independent mechanisms, likely involving granzyme B-induced mitochondrial damage or the activation of surrogate executioners like caspase-7 [5,6,7].

Natural Killer (NK) cells are fundamental players in the innate immune response against oncogenic transformation. They operate via a “ready-to-kill” status that does not require prior antigen-priming or MHC restriction. NK cells exert cytotoxicity through multiple coordinated mechanisms, including: (i) the release of lytic granules containing perforin and granzymes; (ii) the induction of apoptosis via death ligands such as FasL and TRAIL; (iii) antibody-dependent cellular cytotoxicity (ADCC); and (iv) the secretion of pro-inflammatory cytokines like IFN-γ and TNF-α. Their activity is governed by a delicate balance between activating receptors (e.g., NKG2D, NKp30, NKp46) and inhibitory signals [8,9,10,11].

The persistence and recurrence of breast tumors are often linked to breast cancer stem cells (bCSCs), characterized by a CD44^+^CD24^−^/dim phenotype [12,13,14]. CSC enrichment represents a well-established mechanism of immune escape, occurring through the downregulation of classical HLA-I molecules and the upregulation of immune-inhibitory ligands such as PD-L1 and PD-L2 [15,16]. Furthermore, the CD44 standard isoform (CD44s) has been shown to activate the PDGFRβ/STAT3 signaling cascade, which is critical for maintaining stemness, cellular proliferation, and survival [12]. Importantly, CSCs exhibit enhanced resistance to apoptosis, metabolic flexibility, and reduced sensitivity to NK-cell-mediated killing compared to their differentiated counterparts [17,18,19].

During cancer treatment, patients frequently receive concomitant medications that may inadvertently modulate anti-tumor immunity. Paracetamol (acetaminophen, APAP) is the most frequently prioritized analgesic for managing cancer-related pain, which affects approximately 44.5% of patients [20]. While widely considered safe, emerging evidence indicates that APAP may exert significant immunomodulatory effects [21,22]. Clinical studies demonstrate that concomitant APAP use is associated with shorter overall survival (OS) and progression-free survival (PFS) in patients treated with immune checkpoint inhibitors (ICIs). Mechanistically, APAP has been reported to suppress IFN-γ production and promote the expansion of regulatory T cells (Tregs), potentially blunting anti-tumor immunity [21,23]. However, its direct effects on cancer cell susceptibility to NK-mediated cytotoxicity remain poorly characterized.

In contrast, Metformin (MET), a first-line treatment for type 2 diabetes, has been repurposed as a potential anti-cancer agent [24,25,26,27,28]. MET inhibits mitochondrial complex I, leading to AMPK activation and subsequent suppression of the mTORC1 pathway, which limits cell growth and induces apoptosis in cancer cells [29,30,31,32,33]. Paradoxically, MET has also been shown to enhance NK cell activity by upregulating activating receptors and increasing IFN-γ production [34]. Recent evidence further supports the immunometabolic role of metformin in modulating NK and NKT cell responses in breast cancer [35]. Additionally, MET targets the CSC population by reducing the CD44^+^/CD24^−^/dim ratio and interfering with stemness signaling [28,30,36,37]. Despite these promising anticancer properties, the clinical efficacy of MET as a monotherapy remains modest, and its impact on the immune susceptibility of surviving tumor cells requires further investigation [27,28].

Despite the ubiquity of APAP and MET in clinical care, their combined influence on tumor immune escape mechanisms—particularly the evasion of NK surveillance by CSCs—remains poorly understood. This knowledge gap is critical, as drug-induced selection of immune-resistant phenotypes could inadvertently compromise both natural immunosurveillance and the efficacy of immunotherapeutic interventions. Therefore, this study was conducted to evaluate the effects of APAP and MET on the growth, phenotype, and NK surveillance evasion potential of breast cancer cells, with particular emphasis on their capacity to enrich for immune-resistant CD44^+^CD24^−^ stem-like cell (hereafter referred to as CSC-like or CSC-enriched cell) subpopulations and modulate the expression of key immune regulatory ligands.

## 2. Results

### 2.1. Drug-Induced Effects on MCF-7 Growth and Morphology

#### 2.1.1. Growth Inhibition and IC_50_ Determination for APAP and MET

**Figure 1:**
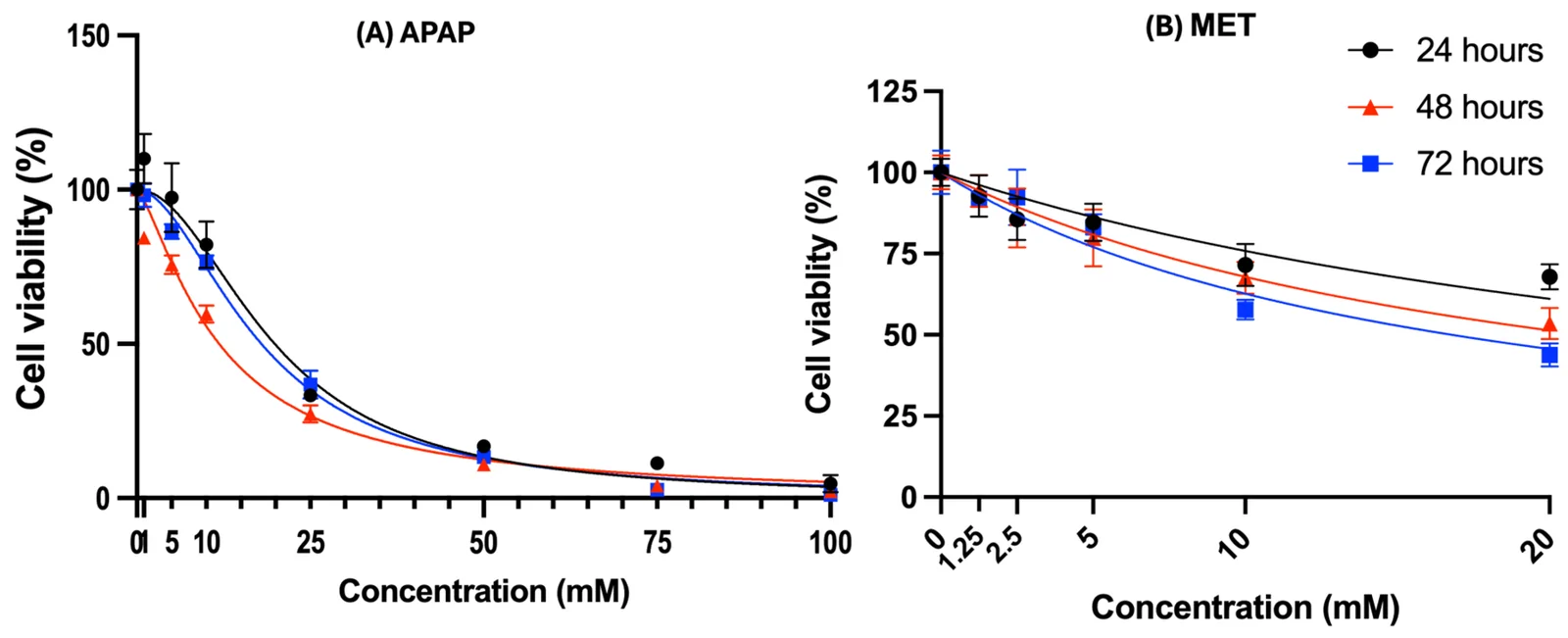
Inhibitory effect of APAP (**A**) and MET (**B**) on the proliferation of MCF-7 cells. (**A**) APAP exhibited time-dependent cytotoxicity toward MCF-7 cells, with IC_50_ values of 19.88 mM (24 h), 11.86 mM (48 h), and 17.96 mM (72 h). Based on these results, 10 mM was selected for subsequent assays. (**B**) MET reduced MCF-7 viability in a time- and dose-dependent manner, with IC_50_ values of 31.40 mM (24 h), 21.11 mM (48 h), and 16.79 mM (72 h). The 48-h IC_50_ (≈20 mM) was selected for subsequent experiments (n = 3).

The half-maximal inhibitory concentration (IC_50_) of both drugs was evaluated at three time points: 24 h, 48 h, and 72 h. For APAP, the IC_50_ values were 19.88 mM, 11.86 mM, and 17.96 mM at the respective time points (Figure 1A). For MET, treatment of MCF-7 cells yielded IC_50_ values of 31.40 mM, 21.11 mM, and 16.79 mM at 24, 48, and 72 h, respectively (Figure 1B). IC_50_ values did not show a strictly monotonic decrease over time for either drug, possibly reflecting adaptive cellular responses. Two-way ANOVA analysis indicated that cell viability was dependent on both treatment duration and drug concentration for both compounds.

Based on these determinations, a concentration of 10 mM (slightly below the 48-h IC_50_ of 11.86 mM) was selected for subsequent APAP experiments. For MET, the 48-h IC_50_ value (21.11 mM) was rounded to 20 mM to facilitate drug preparation, which may also be consistent with locally achievable concentrations reported previously [38].

#### 2.1.2. Morphological Alterations

Prior to co-culture experiments, the phenotypic quality of expanded NK cells was assessed by flow cytometry. The proportion of conventional NK cells (CD3^−^CD16/56^+^) was 59.3%, while T-NK cells (CD3^+^CD16/56^+^) accounted for 9.01% of the population. For simplicity, the expanded product is referred to as “NK cells” throughout the manuscript unless otherwise specified. Together, these two cytotoxic lymphocyte subsets accounted for 68.31% of the total cell population and are expected to contribute substantially to the cytotoxic activity of the expanded cell product, exceeding the commonly accepted quality threshold of ≥50% conventional NK cells for in vitro-expanded NK-cell products (Figure S1).

**Figure 2:**
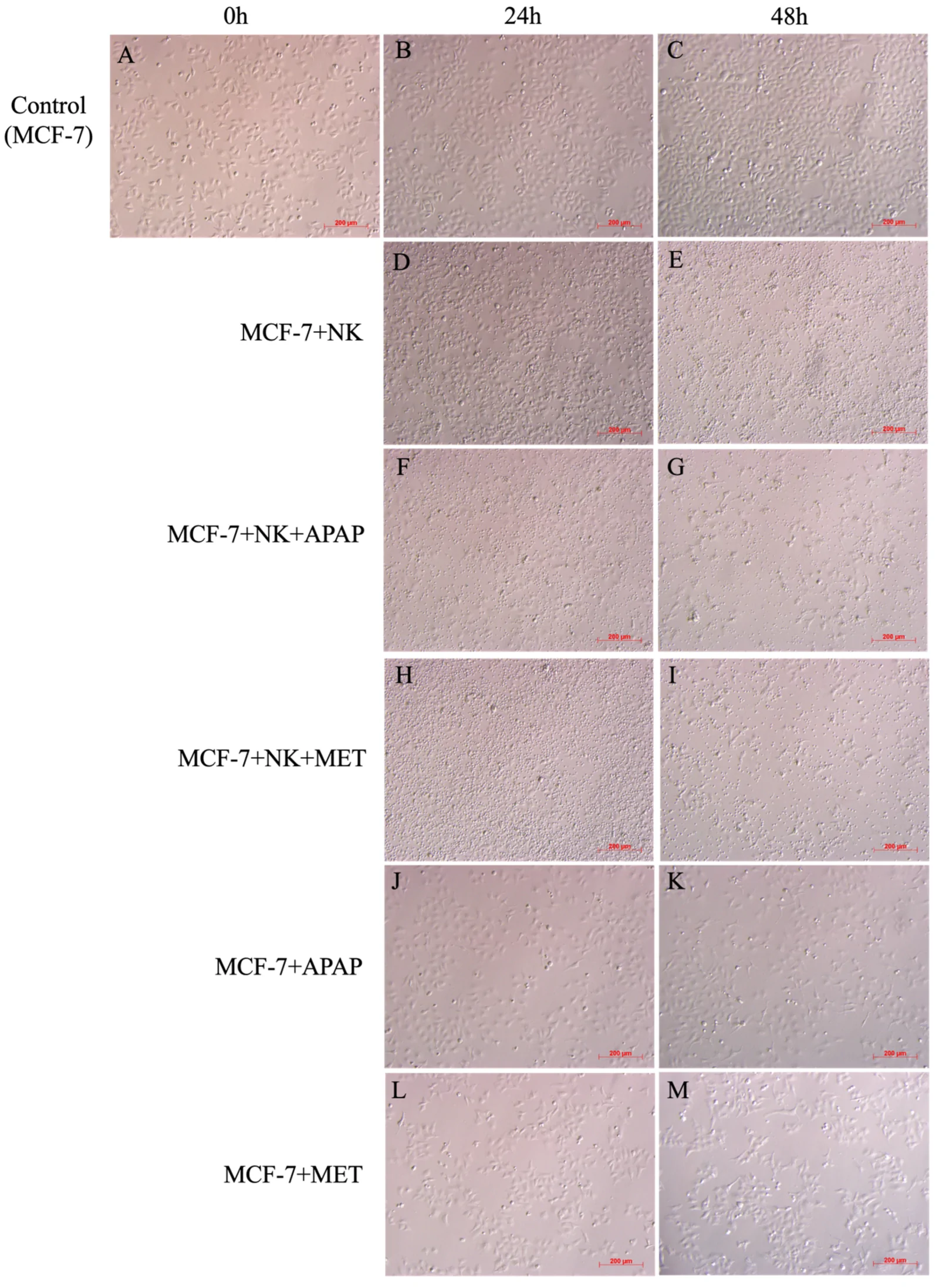
Morphological alterations and density changes of MCF-7 cells during co-culture with NK cells in the presence or absence of APAP and MET. Representative phase-contrast inverted microscopy images showing time-dependent morphological alterations and cell density changes of MCF-7 breast cancer cells under various treatment conditions at 0 h, 24 h, and 48 h. (**A**–**C**) Untreated MCF-7 control cells at 0 h (**A**), 24 h (**B**), and 48 h (**C**), displaying characteristic polygonal, squamous-like epithelial carcinoma morphology with smooth cell membranes and continuous confluent monolayer growth over time. (**D**,**E**) MCF-7 cells co-cultured with expanded NK cells (E:T ratio 20:1) at 24 h (**D**) and 48 h (**E**), showing a prominent reduction in tumor cell density, cell flattening, and detachment consistent with NK-mediated cytotoxicity. (**F**,**G**) MCF-7 cells co-cultured with NK cells and 10 mM APAP at 24 h (**F**) and 48 h (**G**), exhibiting decreased cell density alongside drug-induced morphological elongation. (**H**,**I**) MCF-7 cells co-cultured with NK cells and 20 mM MET at 24 h (**H**) and 48 h (**I**), showing reduced cell confluence with persistent spindle-like cellular alterations. (**J**,**K**) MCF-7 cells treated with 10 mM APAP alone at 24 h (**J**) and 48 h (**K**), demonstrating growth inhibition and transition toward an elongated, fibroblast-like phenotype compared with untreated controls. (**L**,**M**) MCF-7 cells treated with 20 mM MET alone at 24 h (**L**) and 48 h (**M**), showing reduced cell density and prominent spindle-shaped morphological changes over time. Scale bars = 200 µm (red bars at the bottom right of each panel). Images are representative of three independent biological experiments (n = 3).

Inverted microscopy revealed distinct morphological changes and differences in adherent cell density across treatment conditions (Figure 2). In the control group (MCF-7 alone), cells nearly covered the entire culture surface by 48 h, maintained their characteristic squamous-like epithelial carcinoma morphology with smooth, bright membranes, and exhibited frequent mitotic figures. Upon NK cell co-culture, cancer cell density markedly decreased by 48 h, and the remaining cells appeared more elongated, consistent with cell death observed in cytotoxicity assays.

Treatment with APAP or MET alone reduced cell density compared to untreated controls at 24 h, and cells exhibited elongated, fibroblast-like morphology. When NK cells were combined with APAP or MET, cell density was further slightly reduced at 48 h compared to drug treatment alone, although the morphological changes induced by the drugs persisted.

### 2.2. Reduced Susceptibility of MCF-7 Cells to NK Killing Following Drug Treatment

#### 2.2.1. Decreased NK-Mediated Cytotoxicity (7-AAD^+^ Cells) in the Presence of APAP or MET

**Figure 3:**
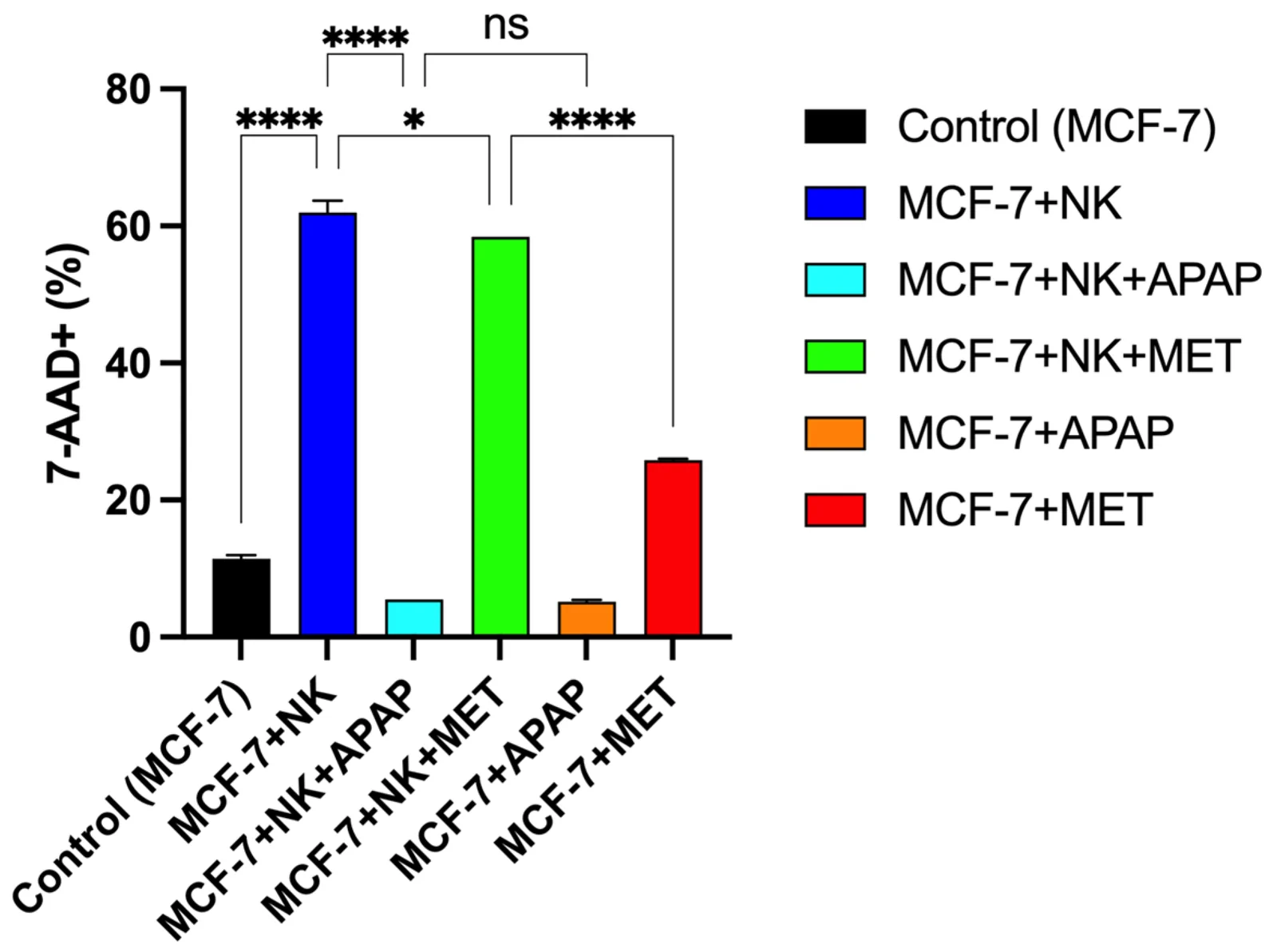
Assessment of MCF-7 cell death during co-culture with NK cells, with or without APAP and MET. Among the residual adherent tumor-cell population recovered after co-culture, 7-AAD staining showed that NK cells (20:1) strongly increased MCF-7 cell death, while APAP (10 mM) reduced both spontaneous and NK-mediated death. MET increased cell death but did not enhance NK cytotoxicity when combined, with NK+MET showing slightly lower killing than NK alone (n = 3). A *p*-value < 0.05 was considered statistically significant (ns: not significant, * *p* < 0.05, **** *p* < 0.0001).

After 48 h of co-culture, MCF-7 cell viability was assessed using 7-AAD staining (Figure 3). The spontaneous death rate of untreated MCF-7 cells was approximately 11.40 ± 0.56%. Addition of NK cells at a 20:1 effector-to-target (E:T) ratio significantly increased MCF-7 cell death to 61.94 ± 1.74% (*p* < 0.0001).

In contrast, treatment with 10 mM APAP markedly reduced both spontaneous MCF-7 death (5.16 ± 0.27%, *p* < 0.001) and NK-mediated cytotoxicity (5.51% dead cells in NK + APAP co-culture, *p* < 0.01 compared to NK alone). MET treatment alone increased MCF-7 cell death to 25.08 ± 0.20% (*p* < 0.0001 vs. control). However, combining MET with NK cells did not further enhance cancer cell death; the observed death rate was 58.40%, slightly lower than NK treatment alone (61.94 ± 1.74%).

#### 2.2.2. APAP and MET Susceptibility of NK

**Figure 4:**
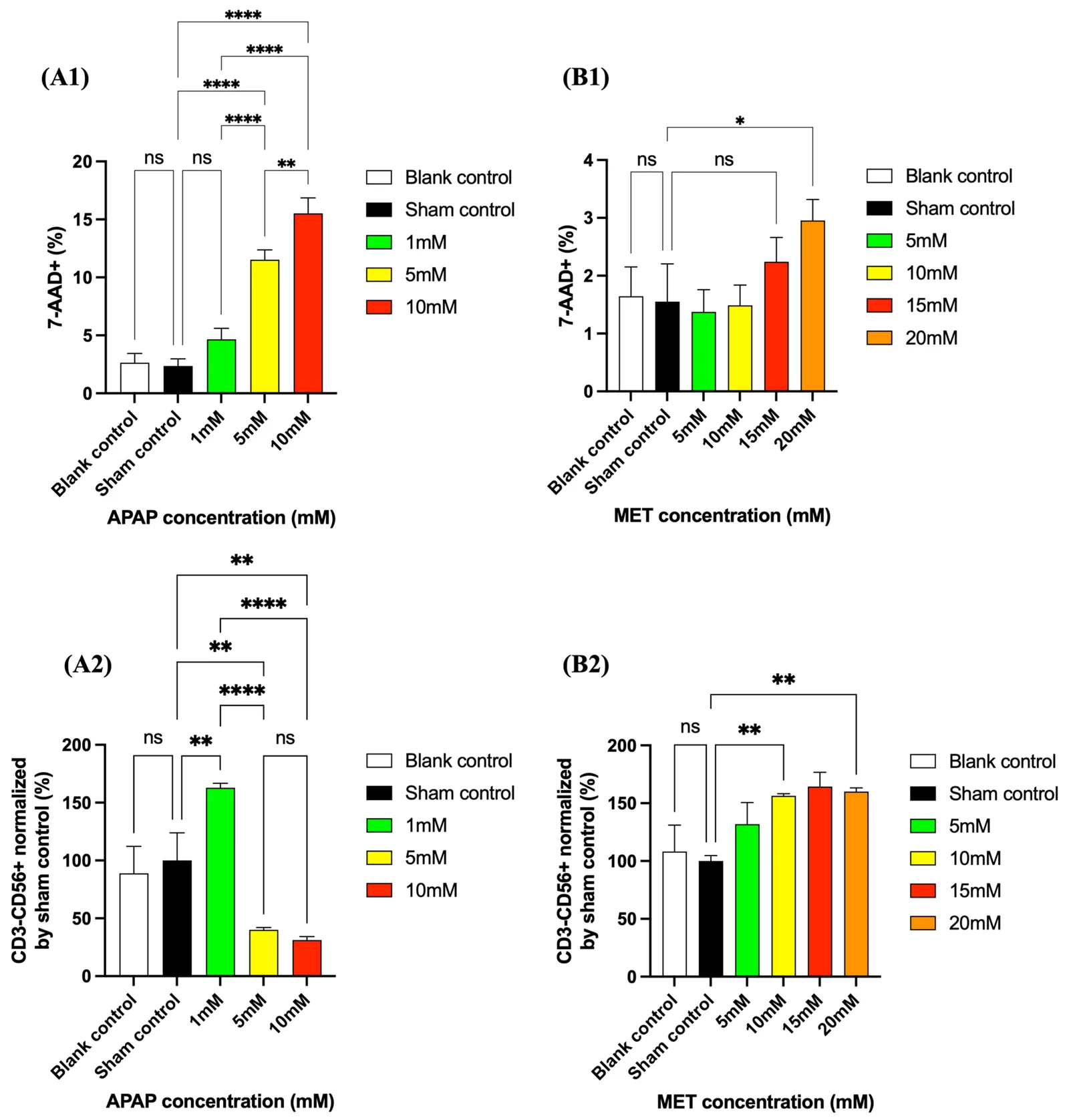
Direct effects of APAP and MET on NK-cell survival and phenotype. (**A1**) Viability of NK cells after 48 h exposure to APAP (1–10 mM). (**A2**) Relative proportion of CD3^−^CD56^+^ NK cells following APAP treatment, normalized to the sham control. (**B1**) Viability of NK cells after 48 h exposure to MET (5–20 mM). (**B2**) Relative proportion of CD3^−^CD56^+^ NK cells following MET treatment, normalized to the sham control. APAP reduced NK-cell viability and the proportion of CD3^−^CD56^+^ cells at concentrations ≥ 5 mM, whereas MET had minimal effects on viability and did not reduce NK-cell phenotype at any tested concentration (n = 3). A *p*-value < 0.05 was considered statistically significant (ns: not significant, * *p* < 0.05, ** *p* < 0.01, **** *p* < 0.0001).

To determine whether APAP and MET directly affect NK cells, expanded NK cells were exposed to increasing concentrations of each drug for 48 h and subsequently analyzed for cell death and NK-cell phenotype (Figure 4).

APAP induced concentration-dependent effects on NK cells. The proportion of 7-AAD^+^ NK cells increased from 2.35 ± 0.63% in the sham control to 4.66 ± 0.95%, 11.52 ± 0.85%, and 15.53 ± 1.33% following exposure to 1, 5, and 10 mM APAP, respectively (Figure 4(A1)). Consistent with this finding, the relative proportion of CD3^−^CD56^+^ cells increased to 162.8 ± 3.8% at 1 mM APAP but declined markedly to 40.15 ± 1.98% and 31.17 ± 3.08% at 5 and 10 mM, respectively (Figure 4(A2)).

In contrast, MET exerted only minor effects on NK cells. The percentage of 7-AAD^+^ cells remained low across all tested concentrations, ranging from 1.37 ± 0.38% to 2.95 ± 0.36% (Figure 4(B1)). Meanwhile, the relative proportion of CD3^−^CD56^+^ cells increased from 100% in the sham control to 131.90 ± 18.69%, 156.50 ± 1.71%, 164.50 ± 12.33%, and 160.10 ± 3.19% at 5, 10, 15, and 20 mM MET, respectively (Figure 4(B2)).

These results indicate that APAP, particularly at 10 mM, directly compromises NK-cell survival and reduces the proportion of CD3^−^CD56^+^ cells, whereas MET has minimal adverse effects on NK-cell viability or phenotype under the conditions used in this study.

#### 2.2.3. Differential NK Susceptibility of CSC-like and Non-CSC Populations

**Figure 5:**
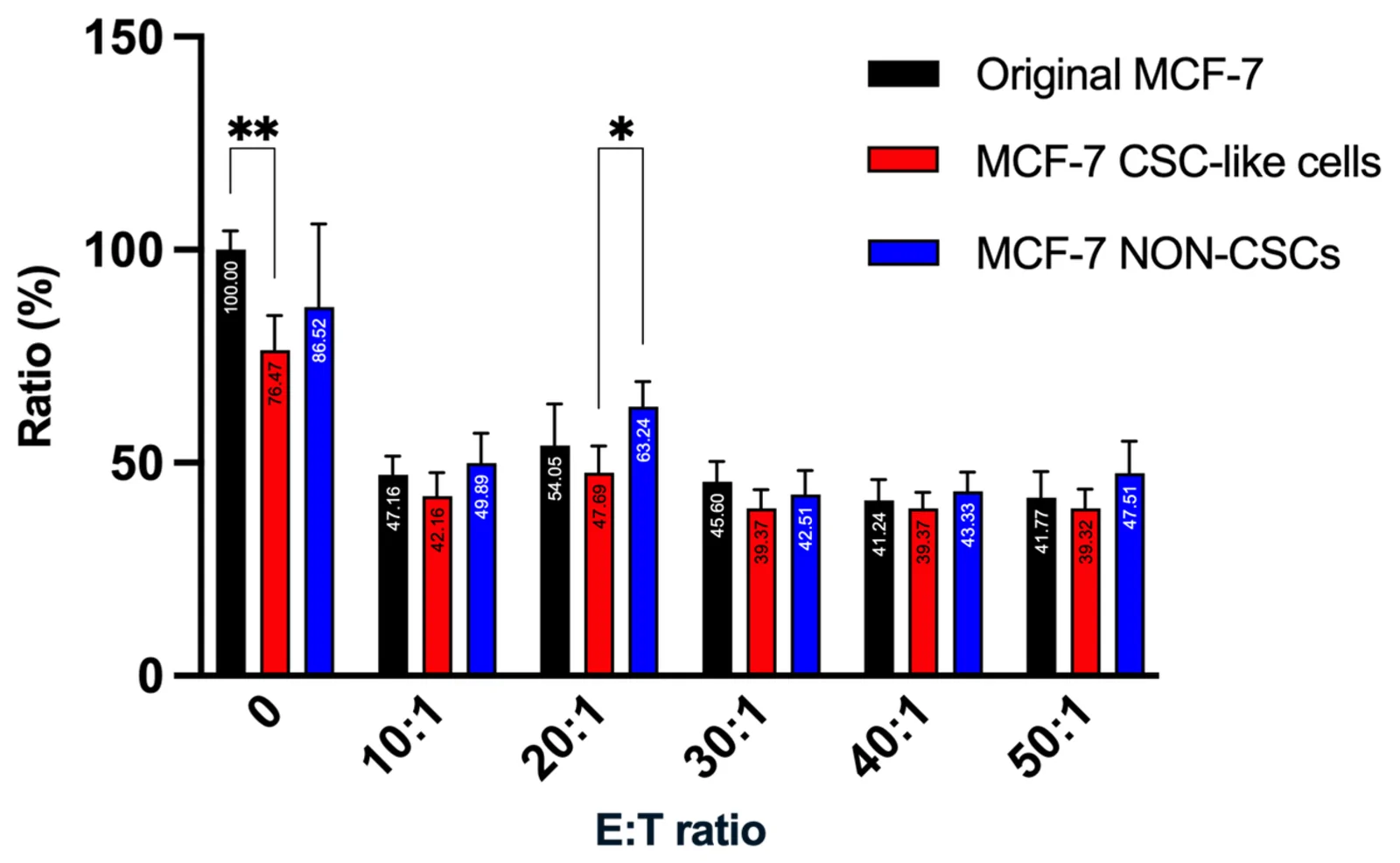
Proliferation capacity of MCF-7 origin, CSC-like cells, and NON-CSCs during co-culture with NK cells across different E:T ratios. Original MCF-7 (CD44^+^/^−^CD24^+^/^−^), MCF-7 CSC-like cells (CD44^+^CD24^−^), and MCF-7 NON-CSCs (CD44^−^CD24^+^/^−^ and CD44^+^CD24^+^). NK cells reduced proliferation of all populations at E:T 10:1, with no further suppression at higher ratios. At 20:1, NON-CSCs showed significantly greater proliferation than CSC-like cells (* *p* < 0.05), distinguishing NK-mediated inhibition between populations (n = 3). A *p*-value < 0.05 was considered statistically significant (* *p* < 0.05, ** *p* < 0.01).

To determine whether the CSC-enriched subpopulation exhibits differential resistance to NK killing, sorted CD44^+^CD24^−^ (CSC-like) and non-CSC (CD44^−^CD24^+^/^−^ and CD44^+^CD24^+^) populations were co-cultured with NK cells at increasing E:T ratios (10:1 to 50:1) for 48 h. After NK cell removal, adherent cancer cell viability was assessed by AlamarBlue assay (Figure 5).

Although substantial biological variation was observed among sorted populations, an E:T ratio of 20:1 provided the clearest discrimination between CSC-like cells and non-CSC susceptibility and was therefore selected for subsequent experiments. Morphological observations by inverted microscopy were consistent with the proliferation data, showing marked reduction in cancer cell density starting at E:T 10:1 across all populations, with no visible differences among higher ratios (Figure S2).

### 2.3. APAP and MET Attenuate NK-Induced Apoptosis and Necrosis

**Figure 6:**
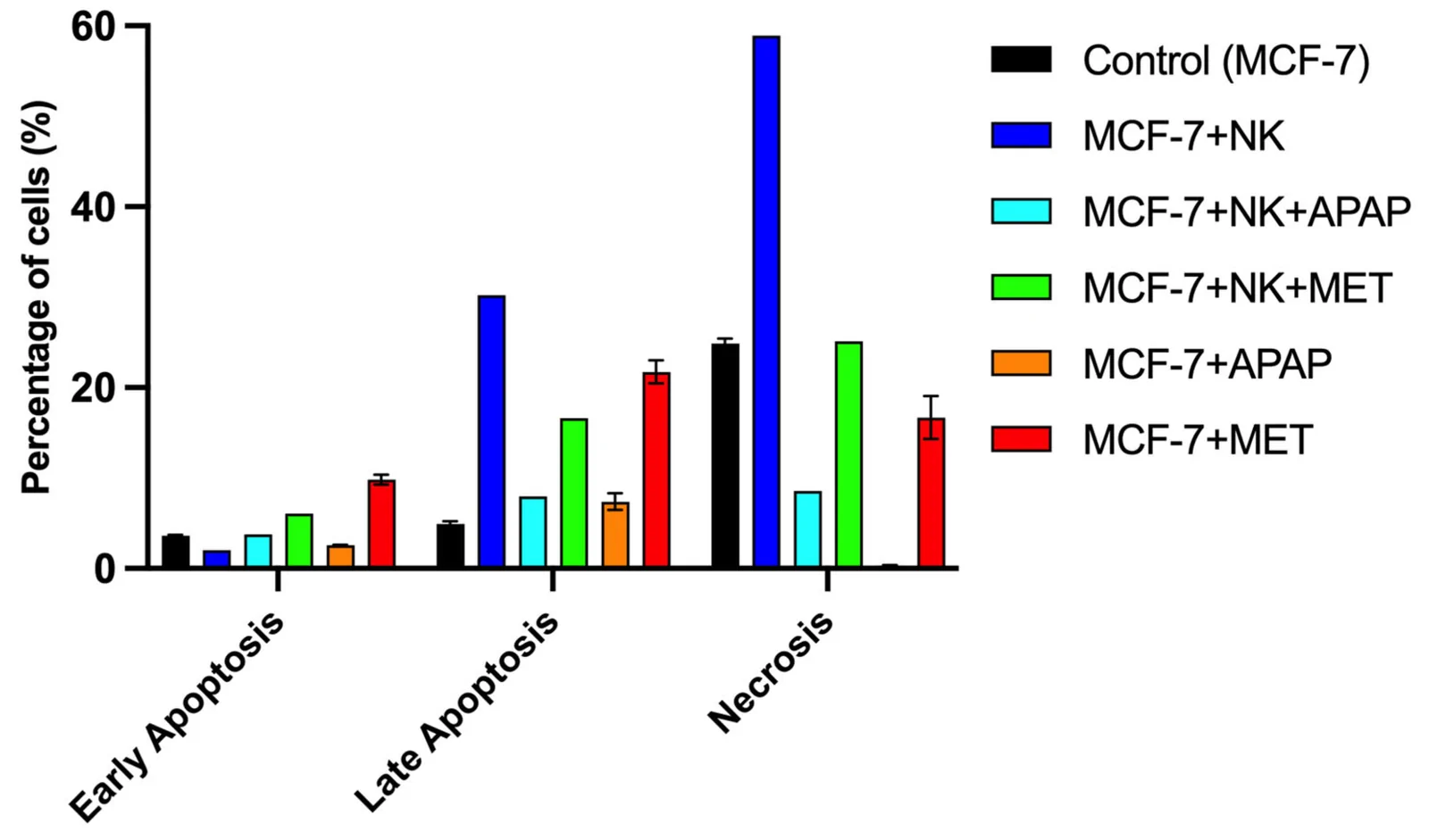
Cell death phenotypes of MCF-7 cells during co-culture with NK cells, with or without APAP and MET. Annexin V/PI analysis demonstrated that co-culture with NK cells markedly increased late apoptosis and necrosis in MCF-7 cells, whereas the addition of APAP or MET significantly reduced both apoptotic and necrotic cell death. For NK-containing groups, residual adherent tumor cells from three independent experiments were pooled prior to staining because cell yields after co-culture were insufficient for reliable flow-cytometric acquisition.

Annexin V-PI staining was performed to characterize the mode of cell death (Figure 6). Co-culture with NK cells alone markedly increased both late apoptosis (from 4.94% in control to 30.20%) and necrosis (from 24.87 ± 0.55% to 58.90%).

Addition of APAP to the NK co-culture dramatically reduced late apoptosis to 7.99% and necrosis to 8.56%, the latter being even lower than the control group. Similarly, MET addition reduced late apoptosis to 16.60% and necrosis to 25.10%.

### 2.4. Cell Cycle Alterations Associated with Drug Treatment and NK Co-Culture

**Figure 7:**
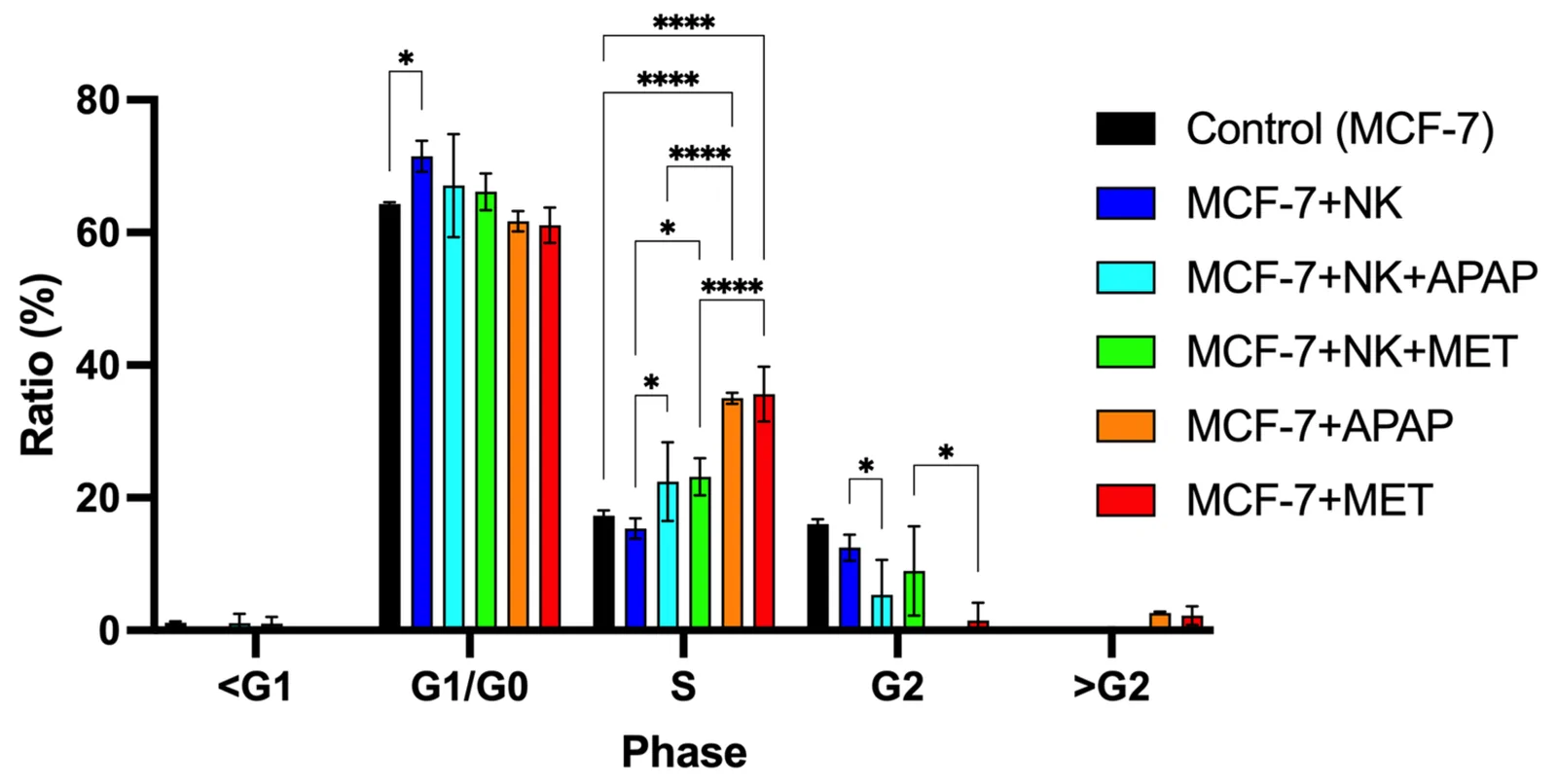
Cell cycle analysis of MCF-7 cells during co-culture with NK cells, with or without APAP and MET. APAP and MET increased the proportion of MCF-7 cells in S phase. Although NK co-culture reduced this effect, S-phase levels remained higher than with NK alone. NK co-culture alone increased G0/G1 arrest (n = 3). A *p*-value < 0.05 was considered statistically significant (* *p* < 0.05, **** *p* < 0.0001).

Cell cycle analysis revealed significant alterations in cell cycle distribution following drug treatment and NK co-culture (Figure 7). Treatment with APAP or MET alone significantly increased the proportion of MCF-7 cells in S phase, reaching 35.00 ± 0.85% and 35.63 ± 4.13%, respectively, compared to 17.30 ± 0.79% in the control group (*p* < 0.0001). When NK cells were added to drug-treated groups, the S phase fraction decreased markedly (APAP + NK: 22.43 ± 5.95%; MET + NK: 23.17 ± 2.80%), yet remained significantly higher than in the NK-alone group (15.37 ± 1.50%, *p* < 0.05).

Notably, the G0/G1 phase proportion was significantly higher in the MCF-7 + NK co-culture (71.50 ± 2.36%) compared to the MCF-7 control group (64.30 ± 0.30%, *p* < 0.05). Additionally, APAP and MET treatment increased the proportion of cells with >G2 DNA content, indicating potential abnormalities in cell division.

### 2.5. Enrichment of Stem-like Cancer Subpopulations Following Drug Treatment and NK Co-Culture

**Figure 8:**
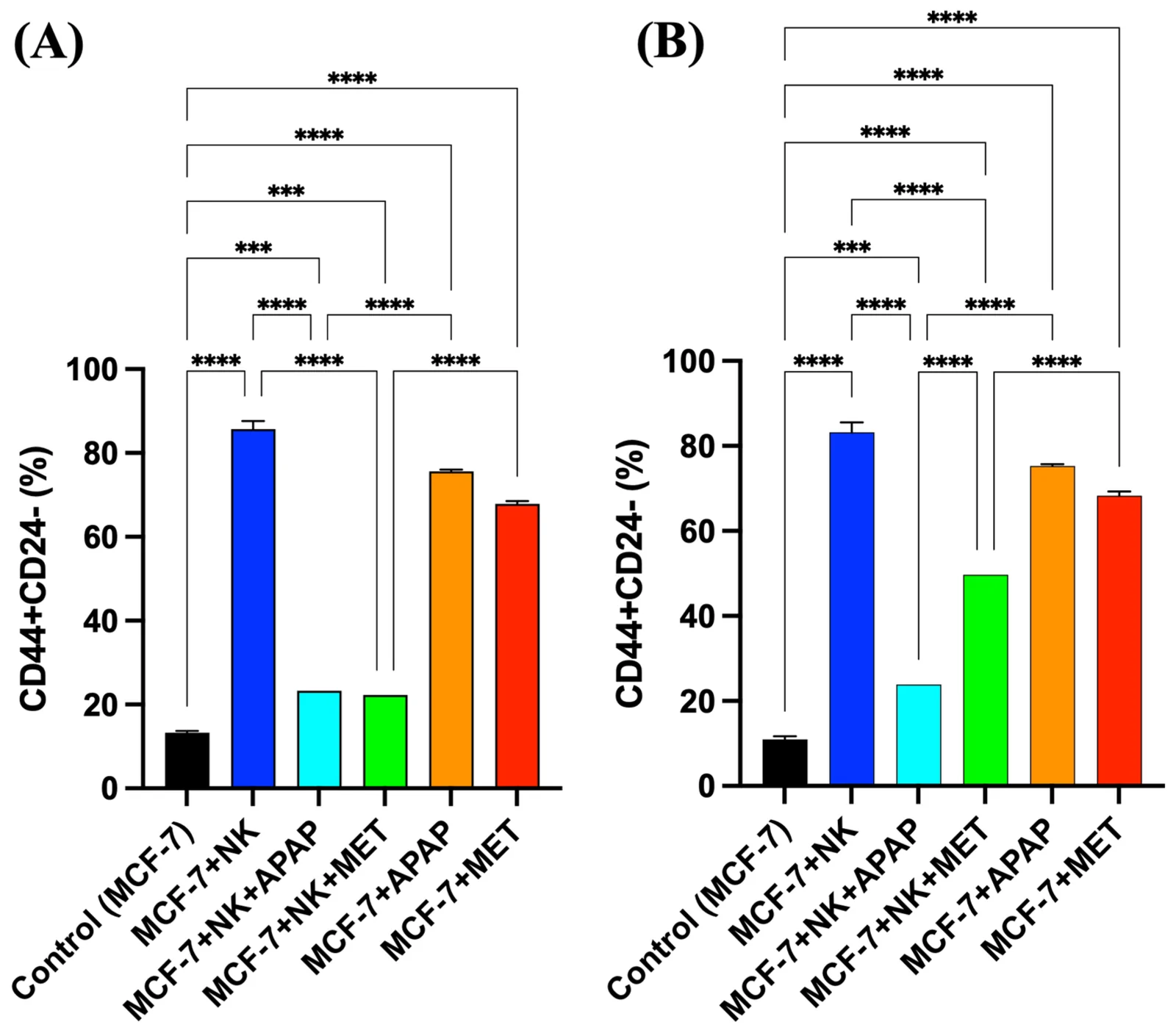
Proportion of CD44^+^CD24^−^ cells in MCF-7 cultures during co-culture with NK cells, with or without APAP and MET. (**A**) Analysis of the total cell population (7-AAD^+^/^−^); (**B**) analysis of the viable cell population (7-AAD^−^). APAP and MET markedly increased the proportion of CD44^+^CD24^−^ cells, whereas NK co-culture (but not NK alone) partially reduced CSC enrichment (n = 3). A *p*-value < 0.05 was considered statistically significant (*** *p* < 0.001, **** *p* < 0.0001).

The proportion of CD44^+^CD24^−^ candidate CSCs among surviving MCF-7 cells was assessed by flow cytometry (Figure 8). APAP treatment dramatically increased the CSC-like population to 75.31 ± 0.42% compared to 11.00 ± 0.67% in the control group (*p* < 0.0001). When NK cells were added to APAP-treated cultures, the CSC-like proportion decreased to 23.89%, but remained significantly higher than control (*p* < 0.001).

MET treatment similarly elevated the CSC-like fraction to 68.31 ± 0.96% (*p* < 0.0001 vs. control). Co-culture with NK cells reduced this to 49.70%, still substantially above control levels (*p* < 0.0001).

Remarkably, NK treatment alone resulted in the highest CSC enrichment (83.2 ± 2.36%, *p* < 0.0001 vs. control), indicating that NK cells preferentially eliminate non-CSC populations, thereby enriching for immune-resistant CSC-like cells. Among all conditions, NK + MET achieved the most favorable balance between overall cancer cell killing and limiting CSC-enriched survivors.

### 2.6. Upregulation of Immune Checkpoint and Immune Evasion-Related Genes

#### 2.6.1. APAP Induces Dose-Dependent Upregulation of Immune-Related Genes

**Figure 9:**
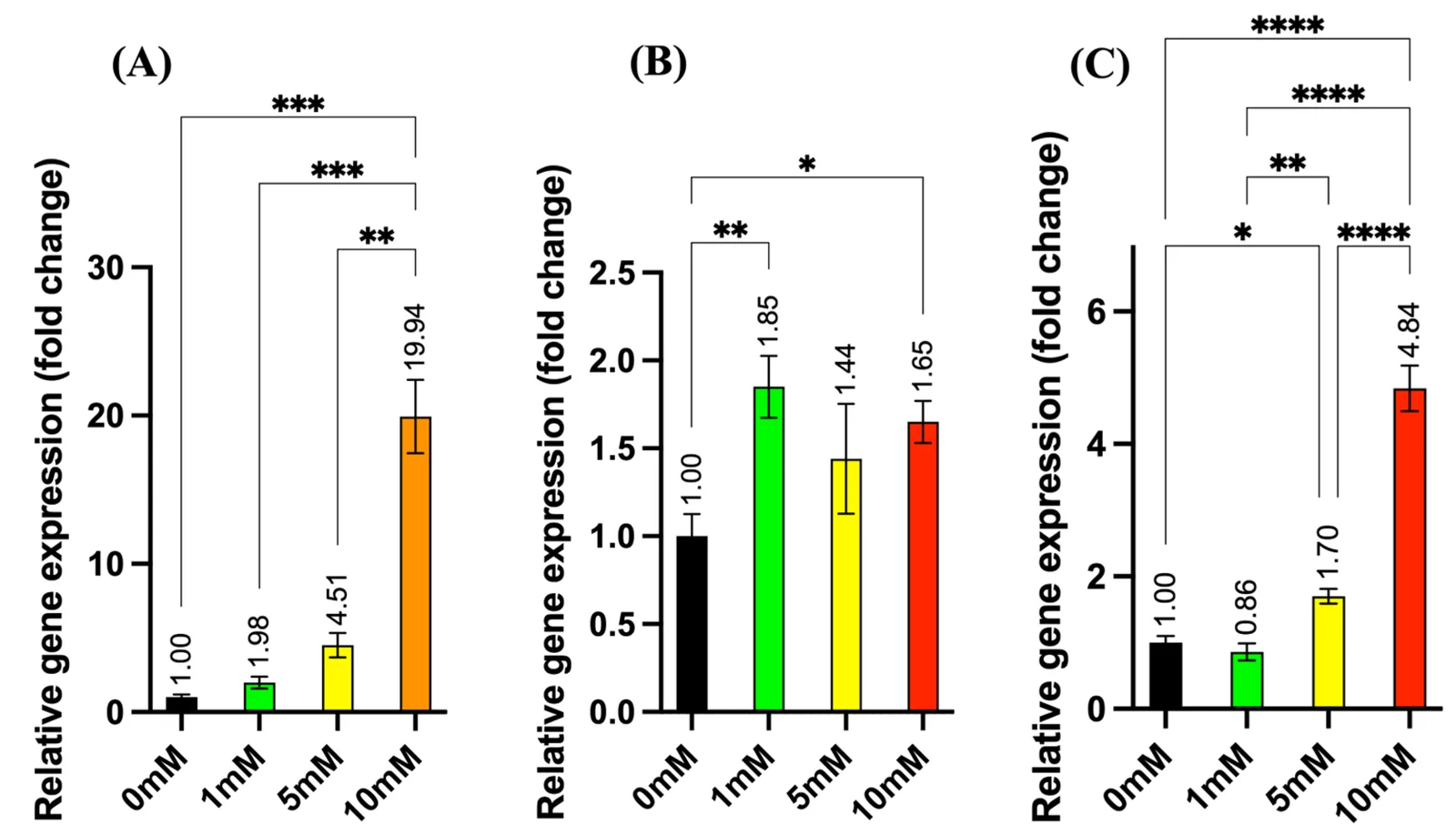

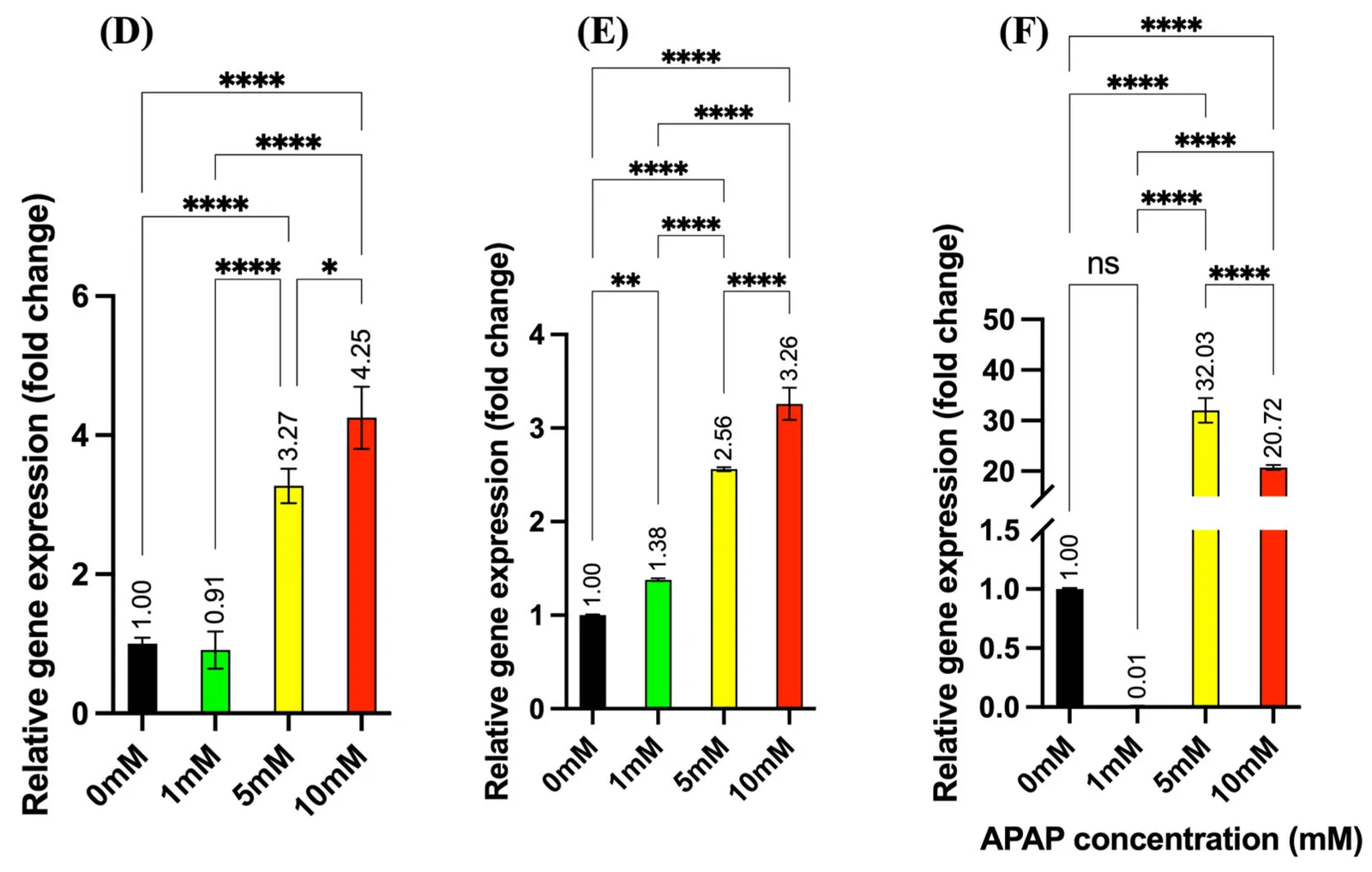
Effects of APAP on the relative expression of immune-evasion-related genes in human breast cancer MCF-7 cells. (**A**) *PD-L1*, (**B**) *PD-L2*, (**C**) *MIC-A*, (**D**) *MIC-B*, (**E**) *CD80*, (**F**) *CD86* (n = 3). A *p*-value < 0.05 was considered statistically significant (ns: not significant, * *p* < 0.05, ** *p* < 0.01, *** *p* < 0.001, **** *p* < 0.0001).

MCF-7 breast cancer cells were treated with APAP for 48 h at concentrations of 0 mM (control, normalized with 0.25% DMSO), 1 mM, 5 mM, and 10 mM. The results showed that APAP induced a dose-dependent upregulation of the examined genes, including *PD-L1/L2*, *MIC-A/B*, and *CD80/86*. Specifically, *PD-L1* expression increased 1.98 ± 0.40-fold (*p* > 0.05), 4.51 ± 0.83-fold (*p* > 0.05), and 19.94 ± 2.48-fold (*p* < 0.001) at 1 mM, 5 mM, and 10 mM, respectively, compared with 0 mM. *PD-L2* expression increased 1.85 ± 0.13-fold (*p* < 0.01), 1.44 ± 0.31-fold (*p* > 0.05), and 1.65 ± 0.12-fold (*p* < 0.05), respectively. *MIC-A/B* expression increased starting at 5 mM: *MIC-A* increased 1.70 ± 0.11-fold (*p* < 0.05) at 5 mM and 4.84 ± 0.34-fold (*p* < 0.0001) at 10 mM. *MIC-B* increased 3.27 ± 0.25-fold and 4.25 ± 0.45-fold (*p* < 0.0001) at 5 mM and 10 mM, respectively. *CD80* expression increased 1.38 ± 0.01-fold (*p* < 0.01), 2.56 ± 0.02-fold, and 3.26 ± 0.17-fold (*p* < 0.0001) at 1 mM, 5 mM, and 10 mM, respectively. *CD86* expression increased 32.03 ± 2.42-fold and 20.72 ± 0.48-fold (*p* < 0.0001) at 5 mM and 10 mM (Figure 9).

#### 2.6.2. MET Induces Dose-Dependent Upregulation of Immune-Related Genes

**Figure 10:**
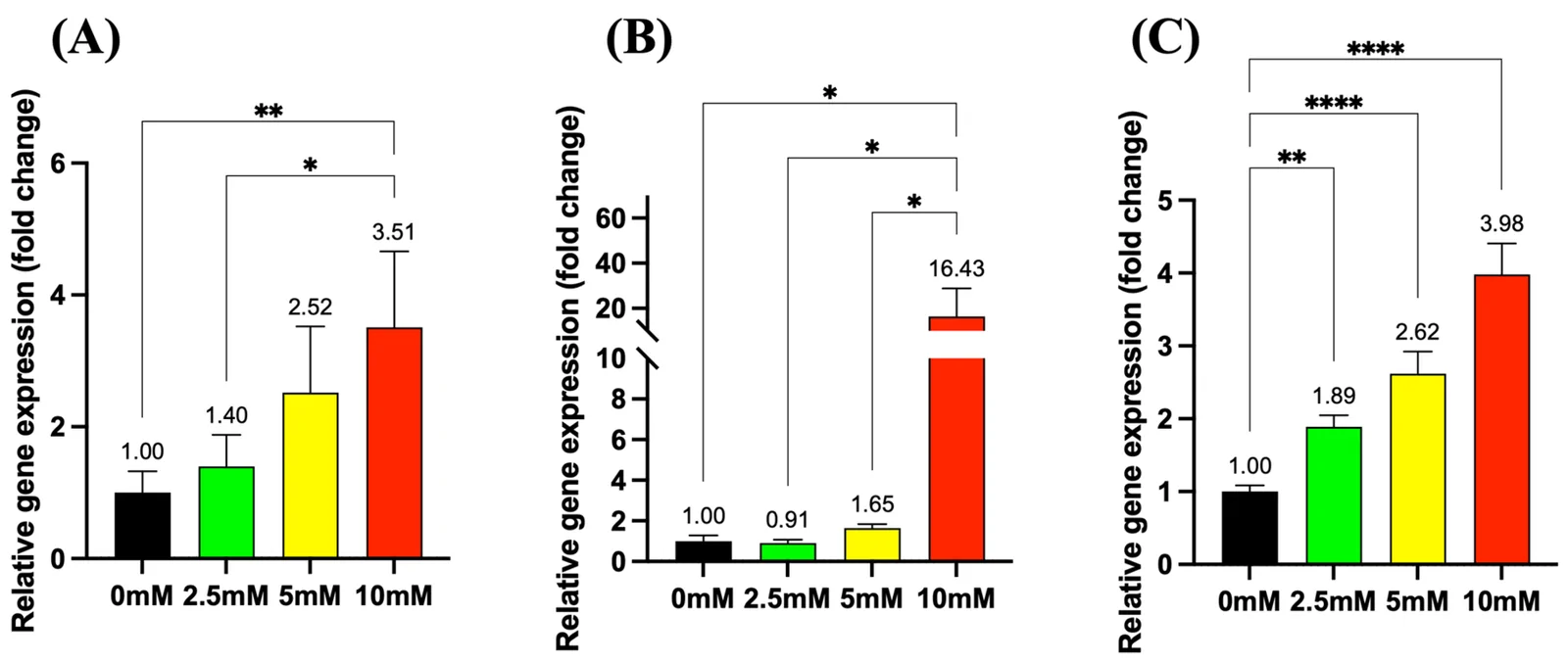

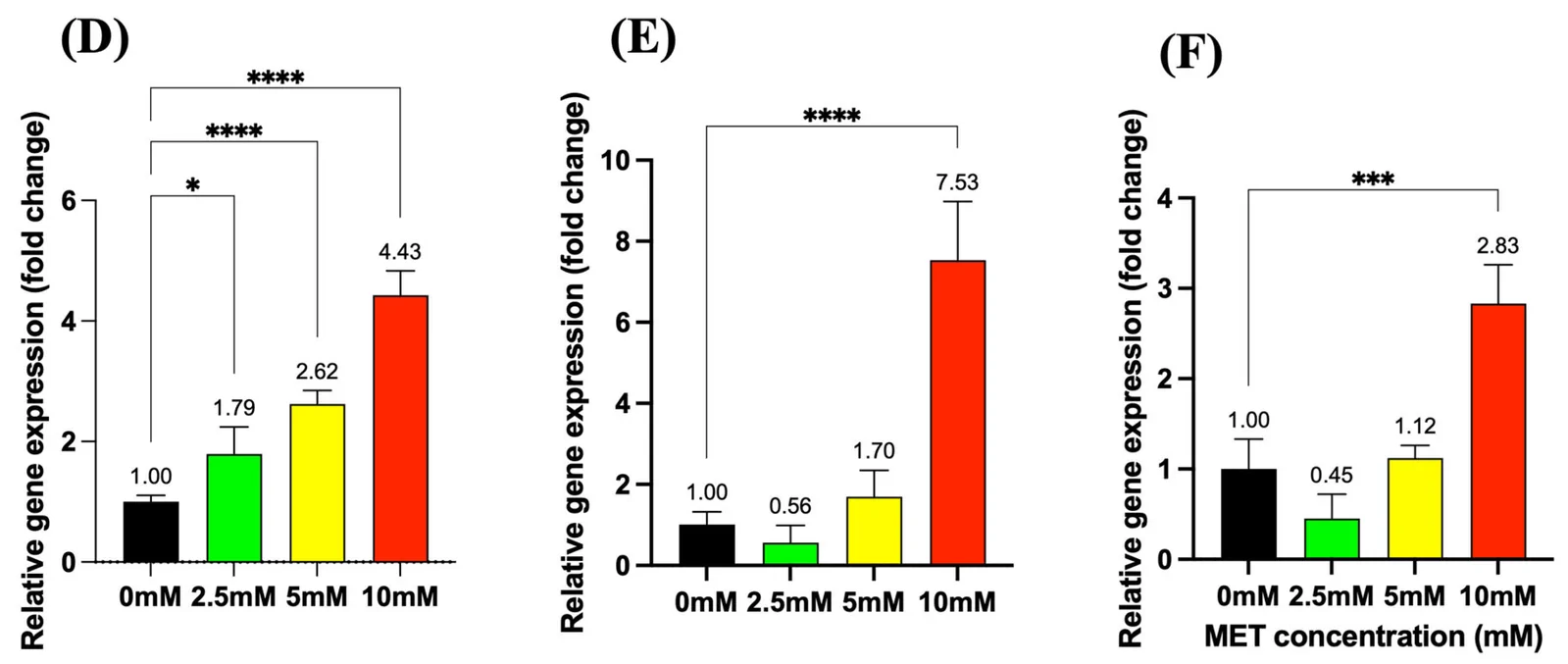
Effects of MET on the relative expression of immune-evasion-related genes in human breast cancer MCF-7 cells. (**A**) *PD-L1*, (**B**) *PD-L2*, (**C**) *MIC-A*, (**D**) *MIC-B*, (**E**) *CD80*, (**F**) *CD86* (n = 3). A *p*-value < 0.05 was considered statistically significant (* *p* < 0.05, ** *p* < 0.01, *** *p* < 0.001, **** *p* < 0.0001).

After treatment with MET at concentrations of 0, 2.5, 5, and 10 mM, total RNA from MCF-7 cells was isolated to evaluate the relative expression of several genes associated with immune evasion in this cell line. Overall, the expression of these genes showed an upward trend. Compared with the control group, *PD-L1* transcript levels increased 1.40 ± 0.477-fold (*p* < 0.05), 2.52 ± 1.002-fold, and 3.51 ± 1.149-fold (*p* < 0.01) at 2.5 mM, 5 mM, and 10 mM, respectively. *PD-L2* expression increased 1.65 ± 0.190-fold (*p* < 0.01) at 5 mM and 16.43 ± 12.442-fold (*p* < 0.01) at 10 mM. *MIC-A* expression increased 1.89 ± 0.157-fold (*p* < 0.01), 2.62 ± 0.301-fold (*p* < 0.0001), and 3.98 ± 0.428-fold (*p* < 0.0001) at 2.5 mM, 5 mM, and 10 mM, respectively. *MIC-B* expression increased 1.79 ± 0.453-fold (*p* < 0.05), 2.62 ± 0.228-fold (*p* < 0.0001), and 4.43 ± 0.400-fold (*p* < 0.0001) at the same respective concentrations. *CD80* expression increased 1.70 ± 0.647-fold at 5 mM and 7.53 ± 1.449-fold (*p* < 0.0001) at 10 mM. Similarly, *CD86* expression began to increase 1.12 ± 0.142-fold and 2.83 ± 0.430-fold (*p* < 0.001) at 5 mM and 10 mM, respectively (Figure 10).

## 3. Discussion

This study reveals a previously unrecognized paradox: two commonly used medications—paracetamol (APAP) and metformin (MET)—while inhibiting breast cancer cell proliferation, simultaneously induce an immune-evasive phenotype that renders surviving cells resistant to Natural Killer cell-mediated cytotoxicity. Our findings suggest that drug-induced growth inhibition and immune susceptibility are mechanistically uncoupled phenomena, with both drugs promoting CSC enrichment, cell cycle alterations, and transcriptional reprogramming toward immune resistance. An important consideration is that the concentrations of APAP (10 mM) and MET (20 mM) used in this study exceed clinically achievable plasma concentrations. These doses were intentionally selected as mechanistic in vitro models to reproduce acute metabolic and oxidative stress within restricted tumor microenvironments, where drug exposure, altered perfusion, local metabolic gradients, and impaired clearance may differ substantially from systemic circulation. Accordingly, the present findings should be interpreted primarily as evidence of potential biological mechanisms rather than direct predictions of clinical pharmacokinetics.

APAP, widely recommended by the WHO for cancer pain management [39], is generally considered safe; however, its potential immunomodulatory effects remain poorly characterized. Conversely, MET, an FDA-approved first-line diabetes medication [25,26], has been repurposed for cancer therapy since the 2010s, though its clinical efficacy as a monotherapy remains modest [27,28]. Our results demonstrate that despite suppressing MCF-7 proliferation (IC_50_: 11.86 mM for APAP; 21.11 mM for MET), neither drug enhanced NK-mediated cytotoxicity. Instead, APAP conferred strong cytoprotection, reducing both spontaneous and NK-induced cell death, while MET—despite triggering intrinsic apoptosis when administered alone—failed to synergize with NK killing upon co-culture.

To determine whether the reduced NK-mediated killing observed in drug-treated co-cultures was attributable to direct effects on NK cells, we exposed expanded NK cells to APAP or MET under the same conditions used in subsequent experiments. The two drugs exhibited distinct effects. MET produced only minimal changes in NK-cell death and did not reduce the relative abundance of CD3^−^CD56^+^ cells, suggesting that the reduced susceptibility of MET-treated MCF-7 cells to NK-mediated cytotoxicity is primarily driven by tumor-cell–intrinsic alterations. In contrast, APAP increased NK-cell death and markedly reduced the proportion of CD3^−^CD56^+^ cells at 10 mM, the concentration used in the co-culture assays. These findings indicate that the diminished NK-mediated killing observed following APAP treatment may result from both direct impairment of NK cells and APAP-induced selection of immune-evasive tumor-cell subpopulations. Importantly, despite their distinct effects on NK cells, both APAP and MET promoted enrichment of CD44^+^CD24^−^ cells and increased expression of multiple immune-evasion–associated genes, supporting a common tumor-cell adaptation mechanism. Collectively, these findings suggest that tumor growth inhibition and immune susceptibility are not necessarily coupled. Also, APAP and MET converge phenotypically in reducing NK-mediated elimination of MCF-7 cells but differ mechanistically, with APAP additionally impairing NK cells directly, whereas MET primarily acts through tumor-cell adaptation.

One of the most striking findings of this study was the marked enrichment of CD44^+^CD24^−^ CSC-like cells following drug treatment, increasing 6.85-fold with APAP (75.31%) and 6.21-fold with MET (68.31%). CSCs are well-established mediators of therapy resistance because of their enhanced resistance to apoptosis, metabolic flexibility, and reduced susceptibility to NK-cell-mediated cytotoxicity [19,40,41,42,43]. The concurrent appearance of fibroblast-like morphology in drug-treated cells might further support EMT induction, which is closely associated with stemness acquisition [44,45].

Despite their low MHC-I expression, CSCs evade NK-cell surveillance through multiple mechanisms, including increased anti-apoptotic signaling, resistance to ADCC, secretion of immunosuppressive factors, altered expression of activating and inhibitory ligands, and shedding of soluble NKG2D ligands [46,47,48,49,50,51]. Consistent with these characteristics, NK cells preferentially eliminated non-CSCs, resulting in marked CSC enrichment (83.2%) in NK-alone cultures. This “therapeutic trap”, in which effective elimination of bulk tumor cells inadvertently enriches highly resistant CSC-like cells, has recently been reported and highlights the need for immunotherapies that directly target the CSC compartment [52]. Interestingly, sorted CSC-like cells did not exhibit greater proliferative capacity than non-CSCs during NK co-culture (Figure 5). Therefore, the marked increase in the CD44^+^CD24^−^ fraction observed after drug treatment is unlikely to result simply from preferential expansion of pre-existing CSC-like cells or elimination of non-CSCs. Instead, these findings are more consistent with therapy-induced phenotypic plasticity, whereby selective pressure imposed by APAP, MET, and NK-cell cytotoxicity reshapes the tumor-cell population toward a stem-like state. Similar therapy-driven dedifferentiation and dynamic interconversion between CSC-like cells and non-CSC states have been increasingly recognized as major mechanisms of tumor adaptation and immune escape.

Combining NK cells with APAP or MET partially reduced drug-induced CSC enrichment, indicating that NK cells remained capable of eliminating a subset of CSC-like cells. Nevertheless, a substantial CSC-like population persisted, potentially because of multiple immune-evasion mechanisms, including HLA-G expression, SOX2/SOX9-mediated regulation, inhibitory NK-receptor engagement, reduced immunogenicity, quiescence, and therapy-induced dedifferentiation of non-CSCs into CSC-like cells [43,52,53].

The cell cycle alterations we observed provide additional mechanistic insight. Both APAP and MET doubled the S phase fraction (to ~35%), suggesting drug-induced S phase accumulation or slowed S phase progression. Upon NK co-culture, S phase fractions decreased but remained elevated relative to NK alone, consistent with persistence of cycling CSC-like populations.

Notably, NK cells alone increased the G0/G1 fraction to 71.50%, indicating induction of quiescence—a known CSC-associated survival mechanism [42]. CSCs have been shown to enter reversible cell cycle arrest to resist conventional therapies [54,55], and similar G0/G1 accumulation has been reported in MCF-7 cells treated with tamoxifen or doxorubicin, with resistant cells subsequently re-entering the cell cycle [56,57]. This quiescence-reactivation cycle may underlie tumor recurrence following seemingly effective therapy.

The distinct cell death profiles induced by APAP and MET warrant discussion. APAP treatment alone did not significantly increase apoptosis, consistent with its well-characterized propensity to induce necrosis rather than apoptosis in hepatocytes [58,59,60,61,62]. However, one study in HEK293 cells reported dose-dependent apoptosis at 5–30 mM APAP [63], suggesting cell-type-specific responses. Low-dose APAP (0.1 mM) has even been reported to stimulate MCF-7 proliferation [64], potentially reflecting CSC expansion rather than true proliferation [42]. Indeed, our data show that APAP’s primary effect is CSC enrichment (6.85-fold) rather than direct killing, explaining the apparent “cytoprotection” observed.

In contrast, MET strongly induced apoptosis when administered alone. This observation might be consistent with its established mechanism of AMPK activation and mTORC1 inhibition [65,66,67,68,69,70]. However, this intrinsic pro-apoptotic activity did not translate to enhanced NK killing, likely due to concurrent selection of immune-resistant CSC-like cells. Allende-Vega et al. [38] similarly reported that 2 mM MET barely affected leukemic cell survival during NK co-culture, though our use of 20 mM revealed more potent direct cytotoxicity. The failure of MET to synergize with NK cells underscores the principle that direct killing and immune sensitization are distinct therapeutic endpoints requiring separate optimization.

The transcriptional changes observed in this study provide a plausible molecular explanation for the reduced susceptibility of MCF-7 cells to NK-cell-mediated killing. Both APAP and MET upregulated the transcriptional immune checkpoint ligands *PD-L1* and *PD-L2*, with APAP inducing a 19.9-fold increase in *PD-L1* and MET a 16.4-fold increase in *PD-L2*. Although PD-1 expression is generally restricted to mature NK-cell subsets, it can be aberrantly induced in the tumor microenvironment, allowing PD-1/PD-L1 signaling to suppress NK-cell activity [43,48,51,71,72,73]. CSC-derived exosomal PD-L1 may further reinforce this immunosuppressive effect [71,72].

An apparent paradox was the concurrent upregulation of the activating ligands *MICA* and *MICB*. While increased transcription would theoretically enhance NK-cell recognition, many tumors evade immune surveillance by shedding soluble MICA/B (sMICA/B), which downregulates NKG2D expression and impairs NK-cell function [74,75,76,77,78,79]. Because our analyses were limited to mRNA expression, we could not distinguish membrane-bound from soluble MICA/B, leaving the functional consequence of this transcriptional upregulation unresolved.

Similarly, the biological significance of *CD80* and *CD86* upregulation remains context-dependent. These molecules can stimulate NK cells through CD28 but may also deliver inhibitory signals via CTLA-4, while CD28 expression is frequently low on mature NK cells [80,81,82,83,84,85]. Therefore, despite the coordinated transcriptional upregulation of several immune-evasion–associated genes, mRNA abundance does not necessarily reflect functional protein expression because of post-transcriptional regulation, protein turnover, and ligand shedding. Future studies should therefore validate PD-L1, PD-L2, CD80, CD86, and MICA/B at the protein level and distinguish membrane-bound from soluble forms to define their precise roles in NK-cell resistance.

Synthesizing our findings, we suggest that APAP and MET induce immune-resistant phenotypes through four convergent mechanisms: (i) selective enrichment of CD44^+^CD24^−^ CSC-like cells with intrinsic immune resistance; (ii) S phase accumulation or slowed S phase progression that maintains the population; (iii) transcriptional upregulation of immune checkpoints (*PD-L1/L2*) that might directly inhibit NK cell function; and (iv) potential induction of soluble NKG2D ligands that systemically impair NK activation (Table 1). These mechanisms likely act synergistically, creating a multifaceted barrier to effective immunosurveillance.

Notably, NK cells themselves contribute to this problem by preferentially eliminating non-CSCs. This, combined with the ability of cancer cells to dedifferentiate into a CSC-like state, creates selective pressure that further concentrates the CSC pool. This “immunoediting” phenomenon suggests that even successful immunotherapy may fail to achieve durable responses unless CSC-targeting strategies are incorporated.

Our findings raise important considerations for clinical practice. APAP, despite its safety profile and WHO recommendation for cancer pain management [39], may inadvertently compromise anti-tumor immunity. Recent clinical studies support this concern: concomitant APAP use is associated with shorter overall and progression-free survival in patients receiving immune checkpoint inhibitors [21,22]. Mechanistically, APAP suppresses IFN-γ production and expands regulatory T cells [21]—effects that complement our observations of direct tumor cell reprogramming.

For MET, our data suggest a more nuanced picture. While MET alone enriches CSC-like cells and upregulates inhibitory ligands, MET + NK co-treatment achieved the most favorable balance between tumor killing and CSC-like enrichment among all conditions tested. This raises the possibility that optimizing MET dosing, scheduling, or combination with CSC-targeted agents could unlock its immunotherapeutic potential. Indeed, MET has been shown to enhance NK activity by upregulating activating receptors and increasing IFN-γ production in some contexts [37], suggesting context-dependent effects that merit further exploration.

Several limitations warrant consideration. First, our in vitro design, while essential for mechanistic dissection, cannot capture the complexity of in vivo tumor microenvironments, including stromal interactions, hypoxia, and systemic immune modulation. Also, from a translational perspective, these findings may be relevant to patients receiving NK-cell–based therapies, immune checkpoint inhibitors, or combination immunotherapies. Although APAP and MET are generally considered safe supportive medications, our data suggest that their effects on tumor-cell immune susceptibility warrant further investigation in clinically relevant in vivo models. Validation in orthotopic, syngeneic, or patient-derived xenograft models will be necessary before extrapolating these observations to clinical settings. Second, reliance on a single cell line MCF-7, a luminal A breast cancer model characterized by estrogen receptor positivity and caspase-3 deficiency, limits generalizability to other breast cancer subtypes, particularly HER2-positive or triple-negative breast cancers. Validation in additional breast cancer models, particularly HER2-positive and triple-negative cell lines with intact caspase-3 expression, will be required to determine the generalizability of these findings. Third, our transcriptional analyses require protein-level validation, particularly for distinguishing membrane-bound versus soluble MICA/B and for quantifying PD-L1/L2 surface expression. Fourth, the functional significance of CD80/86 upregulation remains unclear without receptor profiling on NK cells. We also acknowledge that the concentrations of APAP and MET used in this study exceed typical systemic physiological levels. However, these doses were selected to model localized metabolic stress conditions within the tumor microenvironment, where drug accumulation and altered pharmacokinetics may occur. Therefore, the present study should be interpreted primarily as a mechanistic investigation rather than a quantitative pharmacological evaluation of clinically relevant drug exposure.

Continuously, although the direct effects of APAP and MET on NK-cell death and immunophenotype were assessed, the present study did not evaluate additional functional parameters such as degranulation (CD107a), cytokine secretion, perforin/granzyme release, or metabolic reprogramming. Therefore, subtle drug-induced alterations in NK-cell function cannot be excluded and warrant further investigation. In addition, although pooling was required in some NK-treated groups, the apoptosis data should be interpreted as supportive phenotypic evidence rather than precise estimates of biological variability. Lastly, the present study was designed primarily to characterize phenotypic and functional consequences of APAP and MET exposure rather than to define upstream signaling mechanisms. Several pathways previously implicated in CSC maintenance and immune escape, including AMPK/mTOR, STAT3, NF-κB, and epithelial–mesenchymal transition-associated regulators such as Snail and Slug, may contribute to the observed CSC-like cell enrichment and NK resistance.

Future studies should address these gaps through: (i) validation in additional breast cancer models (e.g., triple-negative, HER2+) and effectors (e.g., CD8+, GDT); (ii) protein-level analyses of immune modulators such as PD-L1, PD-L2, MICA/B, CD80, and CD86 expression should be validated by flow cytometry or biochemical validation and assess ligand shedding, particularly for MICA/B, by ELISA; (iii) mechanistic studies implicated in CSC-like cell maintenance and immune escape above will be required to determine whether modulation of these signaling networks directly mediates the observed effects; (iv) in vivo studies using humanized NK mouse models or patient-derived xenografts; (v) exploration of 3D models (mammospheres, organoids) that better recapitulate CSC-like cell biology [86]; (vi) incorporating dose titration under clinically relevant concentrations to better define translational applicability. Future studies should also determine whether prolonged exposure to clinically achievable concentrations of APAP and MET elicits similar effects on cancer cell plasticity, immune-evasion-associated gene expression, and NK-cell susceptibility; and (vii) further dissecting the direct effects of APAP and MET on NK cell functions including degranulation (CD107a), cytokine secretion (IFN-γ), and cytotoxic granule production; (viii) checking whether the observed NK-resistant phenotype can be reversed by metabolic modulators, transporter inhibitors, or combinatorial immunotherapeutic approaches; and (ix) Although the present study demonstrates marked enrichment of the CD44^+^CD24^−^ phenotype after drug treatment, it does not distinguish whether this enrichment results predominantly from selective survival of pre-existing CSC-like cells, therapy-induced phenotypic conversion of non-CSCs, or a combination of both. Lineage-tracing approaches and single-cell transcriptomic analyses will be valuable for resolving these dynamic processes.

## 4. Materials and Methods

### 4.1. Cell Culture

The human breast cancer cell line MCF-7 (Luminal A subtype) was maintained in RPMI 1640 medium (Gibco, Grand Island, NY, USA; Cat# 61870-036) supplemented with 10% fetal bovine serum (FBS; Gibco, Cat# A5256701) and 1× antibiotic-antimycotic solution (Gibco, Cat# 15240-062). Cells were cultured in a humidified incubator at 37 °C with 5% CO_2_, and the culture medium was renewed every 2 days. Upon reaching 80–90% confluence, cells were passaged at a density of 1 × 10^4^ cells/cm^2^ using TrypLE Express (Gibco, Cat# 12604021).

### 4.2. Isolation, Expansion, and Characterization of NK Cells

#### 4.2.1. NK Cell Isolation and Expansion

Peripheral blood (15 mL) was collected from healthy donors into MNC Cell Collection tubes containing ACD-A anticoagulant, following approval by the institutional ethics committee. All donors provided written informed consent prior to blood collection, acknowledging the procedure’s risks and benefits.

Mononuclear cells (MNCs) were isolated by density gradient centrifugation at 1500× *g* for 15 min. The interphase containing MNCs was collected, washed with Washing Buffer, and centrifuged at 800× *g* for 10 min. The resulting cell pellet was resuspended in NKCult D0–4 medium (Regenmedlab, Ho Chi Minh City, VN; Cat# 299) and transferred into a 500 mL NKCult culture bag for static incubation at 37 °C, 5% CO_2_ for 5 days without manipulation.

On day 5, adherent cells were detached by gentle agitation and transferred to a 1 L NKCult expansion bag. NK cell expansion was continued in NKCult D5–21 medium at 37 °C, 5% CO_2_, with 100 mL of fresh medium added every 2 days (days 5, 7, 9, 11, and 13). On day 15, cells were harvested aseptically, pelleted by centrifugation at 800× *g* for 10 min, and subjected to quality control and functional assays.

#### 4.2.2. Immunophenotypic Characterization

Expanded NK cells (0.5–2 × 10^6^) were washed twice with phosphate-buffered saline (PBS; 800× *g*, 5 min) and resuspended in staining buffer (PBS containing 0.1% bovine serum albumin). Cells were stained with 20 µL of BD Multitest™ 6-Color TBNK reagent (BD Biosciences, San Jose, CA, USA; Cat# 644611) for 20 min at room temperature in the dark. After two washes with staining buffer, cells were resuspended in 200–500 µL of staining buffer and analyzed using a BD FACS Melody flow cytometer (BD Biosciences). NK cells were defined as the CD3^−^CD56^+^CD16^+^ population. A minimum threshold of ≥50% NK cells was applied as a quality control criterion for subsequent experiments.

#### 4.2.3. Effect of APAP and MET on NK-Cell Viability and Phenotype

To evaluate whether APAP or MET directly affect NK-cell viability or phenotype, expanded NK cells were cultured for 48 h in complete NK medium supplemented with APAP (1, 5, or 10 mM) or MET (5, 10, 15, or 20 mM). Sham controls received equivalent volumes of vehicle (1% DMSO for APAP and sterile distilled water for MET), while blank controls received culture medium alone.

Cell viability was determined using flow cytometry after staining with 7-AAD. To assess NK-cell phenotype, cells were stained with CD3 FITC and CD56 APC (BD Biosciences) and analyzed by flow cytometry. The proportion of CD3^−^CD56^+^ NK cells was normalized to the corresponding sham control and expressed as relative percentage.

### 4.3. Drug Preparation and Cytotoxicity Assays

#### 4.3.1. Drugs and Reagents

Paracetamol (acetaminophen, APAP; Sigma-Aldrich, St. Louis, MO, USA; Cat# A7085) was dissolved in dimethyl sulfoxide (DMSO) to prepare a stock solution of 5 M. The final DMSO concentration in all APAP-treated groups was normalized to 1% (*v*/*v*) to control for vehicle effects, unless otherwise specified. Metformin hydrochloride (MET; Sigma-Aldrich, Cat# 317240) was dissolved in sterile distilled water to prepare a stock solution of 1 M.

#### 4.3.2. Determination of Half-Maximal Inhibitory Concentration (IC_50_)

MCF-7 cells were seeded into 96-well plates at a density of 2000 cells per well in 200 µL of complete culture medium and allowed to adhere for 24 h. Cells were then treated with increasing concentrations of APAP (0, 1, 5, 10, 25, 50, 75, 100 mM) or MET (0, 1.25, 2.5, 5, 10, 20 mM). Fresh medium containing the respective drugs was replenished every 24 h.

Cell viability was assessed at 24, 48, and 72 h using the AlamarBlue assay (Thermo Fisher Scientific, Waltham, MA, USA; Cat# DAL1025). Briefly, AlamarBlue reagent was added to each well at a final concentration of 10% (*v*/*v*) and incubated for 4 h at 37 °C, 5% CO_2_. Fluorescence was measured using a Beckman Coulter DTX 880 Multimode Microplate Reader at excitation/emission wavelengths of 560/590 nm. Data were normalized to untreated controls, and dose–response curves were generated using nonlinear regression analysis in GraphPad Prism (version 10.4.1). IC_50_ values were calculated for each time point.

#### 4.3.3. Drug Treatment for Subsequent Experiments

Based on the IC_50_ determinations, MCF-7 cells were treated with 10 mM APAP (48 h IC_50_: 11.86 mM) or 20 mM MET (48 h IC_50_: 21.11 mM) for all subsequent co-culture experiments. For dose–response gene expression analyses, cells were treated with APAP (0, 1, 5, 10 mM) or MET (0, 2.5, 5, 10 mM) for 48 h. These concentrations were intentionally selected as mechanistic in vitro models to reproduce acute localized metabolic and redox stress that may occur within restricted tumor microenvironments rather than clinically achievable systemic plasma concentrations. Therefore, the present findings should be interpreted primarily as mechanistic evidence rather than direct pharmacokinetic simulation.

### 4.4. NK Cell Cytotoxicity Assays

#### 4.4.1. Co-Culture Conditions

MCF-7 cells were seeded into T75 culture flasks at a density of 1 × 10^4^ cells/cm^2^ and cultured for 48 h. The medium was then replaced, and expanded NK cells were added at an effector-to-target (E:T) ratio of 20:1 (unless otherwise specified) in the presence or absence of APAP (10 mM) or MET (20 mM). Co-cultures were maintained for 48 h at 37 °C, 5% CO_2_. An E:T ratio of 20:1 was selected based on preliminary optimization experiments (Section 2.2.3), which demonstrated clear discrimination between the NK susceptibility of CSC-enriched and non-CSC populations while maintaining sufficient viable cells for subsequent phenotypic analyses.

#### 4.4.2. Assessment of NK-Mediated Cytotoxicity by 7-AAD Staining

After co-culture, non-adherent NK cells were removed by aspirating the culture medium followed by PBS washing prior to trypsinization of adherent MCF-7 cells. Consequently, the analyzed population consisted predominantly of adherent MCF-7 cells was stained with 7-aminoactinomycin D (7-AAD; BD Pharmingen™, San Diego, CA, USA; Cat# 559925) according to the manufacturer’s protocol. Samples were analyzed by flow cytometry within 1 h, and the percentage of 7-AAD^+^ cells (non-viable) was determined.

#### 4.4.3. Apoptosis and Necrosis Analysis

Apoptotic and necrotic cell death were assessed using the FITC Annexin V Apoptosis Detection Kit I (BD Pharmingen™, Cat# 556547). Briefly, harvested cells were washed twice with cold PBS and resuspended in 1× Binding Buffer at a concentration of 1 × 10^6^ cells/mL. A 100 µL aliquot (1 × 10^5^ cells) was transferred to a 5 mL tube and incubated with 5 µL of FITC Annexin V and 5 µL of propidium iodide (PI) for 15 min at room temperature in the dark. Following incubation, 400 µL of 1× Binding Buffer was added, and samples were analyzed by flow cytometry within 1 h. Cells were categorized as viable (Annexin V^−^/PI^−^), early apoptotic (Annexin V^+^/PI^−^), late apoptotic (Annexin V^+^/PI^+^), or necrotic (Annexin V^−^/PI^+^). Due to the extensive NK-mediated cytotoxicity observed in several NK-containing groups, residual adherent tumor cells recovered from three independent experiments were pooled before Annexin V/PI staining to ensure sufficient event acquisition for flow-cytometric analysis.

### 4.5. Cell Cycle Analysis

Cell cycle distribution was analyzed using the PI/RNase Staining Buffer (BD Pharmingen™, Cat# 550825). MCF-7 cells (1 × 10^6^ per sample) were harvested, washed with PBS, and fixed in ice-cold 70% ethanol at −20 °C for at least 2 h. Fixed cells were washed twice with PBS to remove residual ethanol and resuspended in 0.5 mL of PI/RNase Staining Buffer. Following incubation for 15 min at room temperature in the dark, stained cells were kept at 4 °C and analyzed by flow cytometry within 1 h. Cell cycle distribution (sub-G1, G0/G1, S, and G2/M phases) was determined using FlowJo software v10.10.1 (BD Biosciences).

### 4.6. Isolation and Analysis of Cancer Stem Cell Subpopulations

#### 4.6.1. Rationale for Marker Selection

The CD44^+^CD24^−^ phenotype was selected to identify CSC-like cells, as this population is preferentially found at the invasive edges of tumors and is more representative of the cellular phenotype observed in 2D culture models compared with ALDH1^+^ bCSCs, which are typically enriched in the tumor core.

#### 4.6.2. Flow Cytometric Analysis and Sorting of CSC-like Cells

MCF-7 cells were detached using TrypLE Express (Gibco, Cat# 12604021) and collected by centrifugation at 500× *g* for 5 min. Cell pellets were washed twice with PBS and resuspended in sorting buffer (PBS supplemented with 0.1% BSA). Cells were stained with BD Horizon™ BV421 anti-human CD24 (BD Biosciences, Cat# 562789) and BD Pharmingen™ APC anti-human CD44 (BD Biosciences, Cat# 560890) at 5 µL each for 20 min at 4–8 °C in the dark. After staining, cells were washed twice with sorting buffer (500× *g*, 5 min), resuspended in 1 mL of buffer, and transferred to 6 mL flow cytometry tubes.

Immunophenotypic analysis and fluorescence-activated cell sorting (FACS) were performed on a BD FACS Melody (BD Biosciences) with fluorescence compensation, sequential gating to exclude debris and doublets, and 7-AAD-based viability exclusion. The CD44^+^CD24^−^ population was gated and collected as the CSC-enriched subpopulation. Sorted cells were either resuspended in complete culture medium supplemented with 2× antibiotic–antimycotic for downstream culture or in PBS for subsequent assays.

#### 4.6.3. Differential NK Susceptibility of CSC-like and Non-CSC Populations

Sorted CD44^+^CD24^−^ (CSC-like cells) and non-CSC (CD44^−^CD24^+^/^−^ and CD44^+^CD24^+^) populations were seeded into 96-well plates at a density of 5000 cells per well and allowed to adhere overnight. The following day, NK cells were added at E:T ratios of 0:1, 10:1, 20:1, 30:1, 40:1, and 50:1. After 48 h of co-culture, NK cells were removed by washing at least three times with PBS. The residual viability and proliferation of MCF-7 subpopulations were assessed using the AlamarBlue assay as described in Section 4.3.2. Morphological changes were documented using inverted microscopy at each time point.

### 4.7. Gene Expression Analysis by RT-qPCR

#### 4.7.1. Total RNA Extraction

Total RNA was extracted from 1–10 × 10^5^ MCF-7 cells using the easy-BLUE™ Total RNA Extraction Kit (iNtRON Biotechnology, Seongnam-si, Gyeonggi-do, Republic of Korea; Cat# 17061) according to the manufacturer’s instructions. Briefly, cells were trypsinized, pelleted, and lysed in 1 mL of easy-BLUE™ reagent. Following chloroform extraction and phase separation by centrifugation (13,000 rpm, 15 min, 4 °C), the aqueous phase was collected, and RNA was precipitated with isopropanol (1:1 ratio). The RNA pellet was washed with 70% ethanol, air-dried, and resuspended in 20–50 µL of RNase-free DEPC-treated water. Purified RNA was stored at −80 °C until further analysis. RNA concentration and purity were assessed using a NanoDrop spectrophotometer (Thermo Fisher Scientific).

#### 4.7.2. Quantitative Reverse Transcription PCR (RT-qPCR)

Relative mRNA expression of immune-related genes was quantified using one-step RT-qPCR with the Luna Universal One-Step RT-qPCR Kit (New England Biolabs, Ipswich, MA, USA; Cat# E3005) according to the manufacturer’s protocol. The following genes were analyzed: *PD-L1* (*CD274*), *PD-L2* (*PDCD1LG2*), *MICA*, *MICB*, *CD80*, and *CD86*. Primer sequences are provided in Supplementary Table S1.

Reactions were performed on a LightCycler^®^ 480 II instrument (Roche, Basel, Switzerland) under standard cycling conditions: reverse transcription at 55 °C for 10 min, initial denaturation at 95 °C for 1 min, followed by 40 cycles of denaturation at 95 °C for 10 s and annealing/extension at 60 °C for 30 s. Each reaction was performed in triplicate.

Relative expression levels were calculated using the 2^−ΔΔCt^ method, with *GAPDH* serving as the endogenous reference gene. Results are presented as fold change relative to the untreated control group.

### 4.8. Statistical Analysis

All experiments were performed with at least three independent replicates (n ≥ 3), unless otherwise stated. All quantitative data are presented as mean ± standard deviation (SD) throughout the manuscript. Data normality was assessed using the Shapiro–Wilk test. Because of the small sample size (n = 3), the results of normality testing were interpreted cautiously. Parametric analyses were nevertheless applied consistently because these tests are generally robust for balanced experimental designs and are widely used in similar in vitro studies. Statistical analyses were performed using GraphPad Prism (version 10.4.1). Comparisons between two groups were made using two-tailed Student’s t-test. Multiple group comparisons were performed using one-way or two-way analysis of variance (ANOVA) followed by appropriate post-hoc tests (Tukey’s or Dunnett’s). A *p*-value < 0.05 was considered statistically significant (* *p* < 0.05, ** *p* < 0.01, *** *p* < 0.001, **** *p* < 0.0001).

## 5. Conclusions

In summary, this study demonstrates that APAP and MET, despite inhibiting MCF-7 breast cancer cell proliferation, induce an immune-evasive phenotype characterized by CSC-like cell enrichment, cell cycle alterations, and transcriptional upregulation of immune checkpoints and stress ligands. These changes render surviving cancer cells resistant to NK cell-mediated killing and create a “therapeutic paradox” where growth inhibition does not translate to enhanced immune susceptibility. APAP and MET converged phenotypically in reducing NK-mediated elimination of MCF-7 cells but differed mechanistically, with APAP exerting adverse effects on both NK and cancer cells whereas MET primarily affected tumor-cell characteristics. NK cells further compound this problem by preferentially eliminating non-CSCs, while therapy-induced dedifferentiation of surviving non-CSCs toward a CSC-like phenotype further enriches the resistant CSC pool. Among all conditions tested, MET + NK co-treatment achieved the most favorable balance between tumor killing and limiting CSC-like cell expansion, suggesting potential therapeutic windows worth further investigation. Our findings highlight the need to carefully evaluate immunomodulatory consequences of common medications in cancer patients and support the development of multi-targeted strategies that simultaneously address tumor growth and immune evasion.

## Figures and Tables

**Table 1:** Summary of key phenotypic and molecular alterations in MCF-7 cells following drug treatment and NK cell co-culture.

Parameter	APAP Effect	MET Effect	NK Alone	NK + APAP	NK + MET
Cell death (7-AAD^+^)	↓ (protective)	↑ (toxic alone)	↑↑↑	↓↓↓ (vs. NK)	↓ (vs. NK)
Apoptosis/Necrosis	↓↓↓ both	↓ both	↑↑↑ both	↓↓↓	↓↓
S phase fraction	↑↑	↑↑	↓	↑ (vs. NK)	↑ (vs. NK)
CSC enrichment (CD44^+^CD24^−^)	↑↑↑ (75%)	↑↑↑ (68%)	↑↑↑ (83%)	↑ (24%)	↑↑ (50%)
*PD-L1/PD-L2* expression	↑↑ (PD-L1)	↑↑ (both)	N/A	N/A	N/A
*MIC-A/B* expression	↑↑	↑↑	N/A	N/A	N/A
*CD80/86* expression	↑↑	↑↑	N/A	N/A	N/A

An upward arrow indicates an increase in the parameter, while a downward arrow indicates a decrease.

## Data Availability

Data and materials used and/or analyzed during the current study are available from the corresponding author on reasonable request.

## Supplementary Materials

The following supporting information can be downloaded at: https://www.mdpi.com/article/10.3390/ijms27167211/s1.

## Abbreviations

7-AAD: 7-aminoactinomycin D; ADCC: Antibody-dependent cellular cytotoxicity; AMPK: AMP-activated protein kinase; ANOVA: Analysis of variance; APAP: Paracetamol (acetaminophen); BSA: Bovine serum albumin; CD: Cluster of differentiation; cDNA: complementary DNA; CO_2_: Carbon dioxide; CSC: Cancer stem cell; CTLA-4: Cytotoxic T-lymphocyte-associated protein 4; DEPC: Diethyl pyrocarbonate; DMSO: Dimethyl sulfoxide; DNA: Deoxyribonucleic acid; E:T: Effector-to-target; ELISA: Enzyme-linked immunosorbent assay; EMT: Epithelial-mesenchymal transition; ERK: Extracellular signal-regulated kinase; FACS: Fluorescence-activated cell sorting; FasL: Fas ligand; FBS: Fetal bovine serum; FDA: Food and Drug Administration; FITC: Fluorescein isothiocyanate; GAPDH: Glyceraldehyde-3-phosphate dehydrogenase; HER2: Human epidermal growth factor receptor 2; HLA: Human leukocyte antigen; IC_50_: Half-maximal inhibitory concentration; ICAM-1: Intercellular adhesion molecule-1; ICI: Immune checkpoint inhibitor; IFN-γ: Interferon-gamma; IL: Interleukin; MCF-7: Michigan Cancer Foundation-7; MET: Metformin; MHC: Major histocompatibility complex; MICA: MHC class I polypeptide-related sequence A; MICB: MHC class I polypeptide-related sequence B; MNC: Mononuclear cell; mRNA: messenger RNA; mTORC1: Mechanistic target of rapamycin complex 1; NK: Natural Killer; NKG2D: Natural killer group 2D; OS: Overall survival; PBS: Phosphate-buffered saline; PD-1: Programmed cell death protein 1; PD-L1: Programmed death-ligand 1; PD-L2: Programmed death-ligand 2; PDGFRβ: Platelet-derived growth factor receptor beta; PI: Propidium iodide; PI3K: Phosphoinositide 3-kinase; PFS: Progression-free survival; RNA: Ribonucleic acid; RT-qPCR: Reverse transcription quantitative polymerase chain reaction; SD: Standard deviation; SEM: Standard error of the mean; sMICA/B: soluble MICA/B; SOX2: SRY-box transcription factor 2; SOX9: SRY-box transcription factor 9; STAT3: Signal transducer and activator of transcription 3; TNF-α: Tumor necrosis factor-alpha; TRAIL: TNF-related apoptosis-inducing ligand; Treg: Regulatory T cell; ULBP2: UL16 binding protein 2; WHO: World Health Organization.
