# Supplementary material for: Paracetamol and Metformin Reduce NK-Cell Susceptibility in MCF-7 Breast Cancer Cells in Association with Enrichment of Immune-Evasive CD44+CD24− Stem-like Subpopulations
Source: Int J Mol Sci. 2026 Aug 12;27(16):7211. doi: 10.3390/ijms27167211 (PMC13513177; doi:10.3390/ijms27167211)
Supplement: Supplementary file 1 [file ijms-27-07211-s001.zip › ijms-4449815-supplementary.pdf]

Table S1. List of primers and the primer sequences

Genes	Primer probe	Sequence
PD-L1	Forward	5’-GCTGCACTAATTGTCTATTGGGA-3’
	Reverse	5’-AATTCGCTTGTAGTCGGCACC-3’
PD-L2	Forward	5’- CTCGTTCCACATACCTCAAGTCC-3’
	Reverse	5’- CTGGAACCTTTAGGATGTGAGTG-3’
MIC-A	Forward	5’- CCACCAGGATTTGCCAAGGAGA -3’
	Reverse	5’- CTGCCAATGACTCTGAAGCACC -3’
MIC-B	Forward	5’-CCTAGACTCTACAGCCAGGC-3’
	Reverse	5’-CATGATCCCTTTGCTGGCTC-3’
CD80	Forward	5’-GAAGCAAGGGGCTGAAAAG-3’
	Reverse	5’-GGAAGTTCCCAGAAGAGGTCA-3’
CD86	Forward	5’- AGGACAAGGGCTTGTATCAA-3’
	Reverse	5’- ATTGCTCGTAACATCAGGGA-3’
GAPDH	Forward	5’-TGCTGGCGCTGAGTAGTACGTCG-3’
	Reverse	5’-TGACCTTGGCCAGGGGTGCT-3’

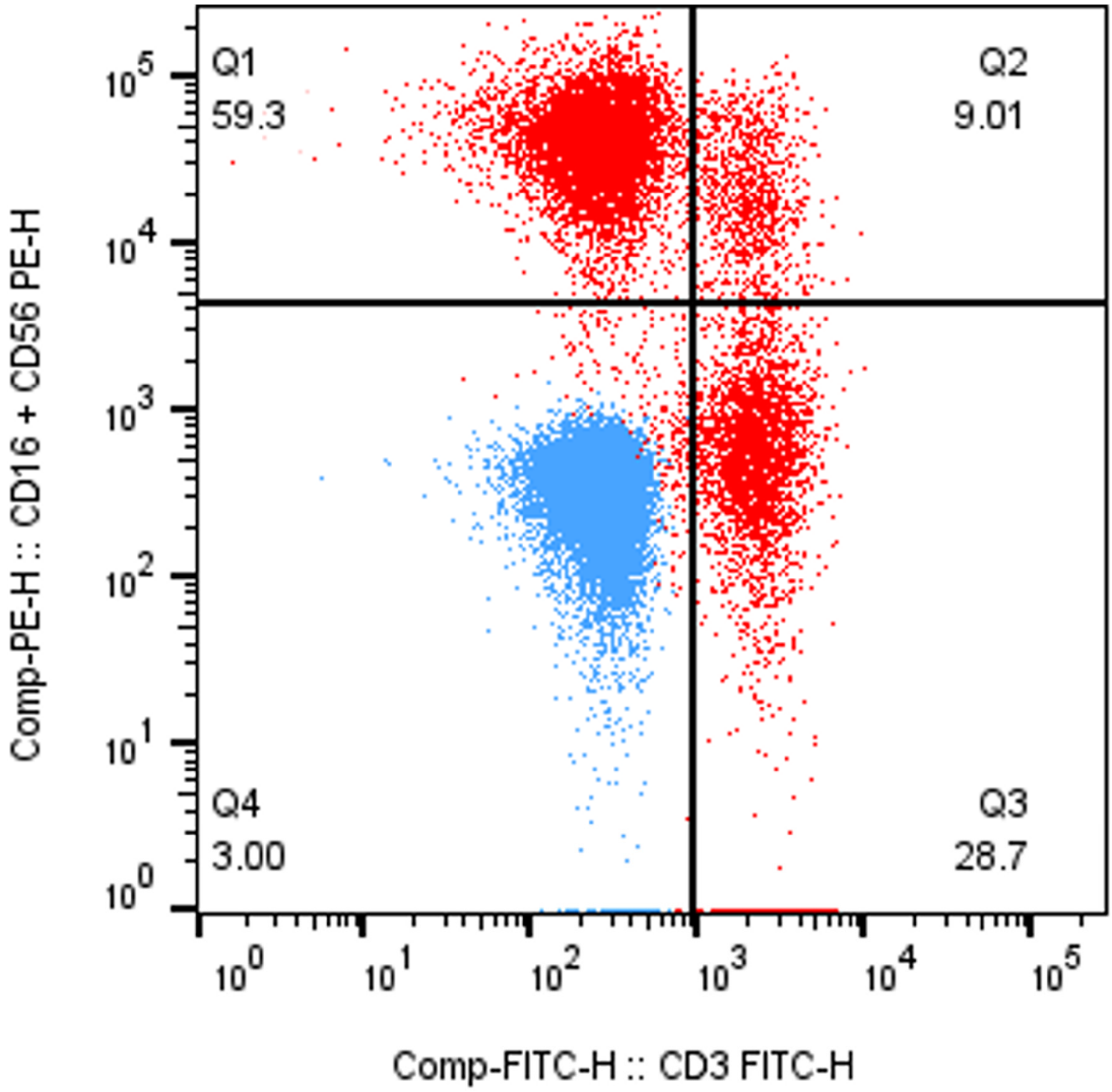

Figure S1. Flow cytometric assessment of NK cell composition prior to co-culture. Flow cytometric analysis showed a high proportion of NK cells ($CD3^-CD16/56^+$, 59.3%) and T-NK cells ($CD3^+CD16/56^+$, 9.01%), resulting in a “combined NK fraction” of 68.31%, exceeding the ≥50% quality threshold for in vitro–expanded NK cell products. Blue: unstained samplel; Red: stained sample.

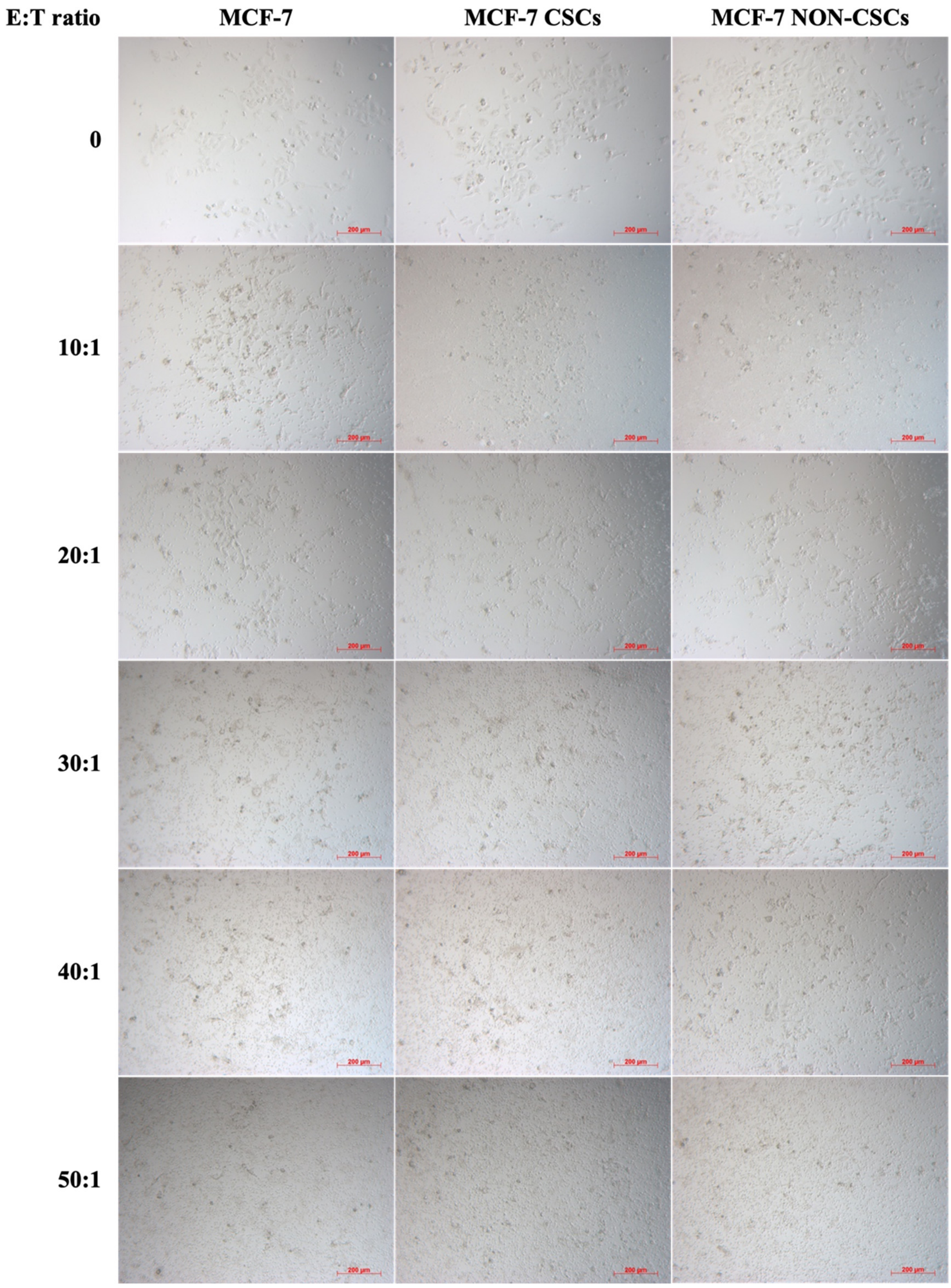

Figure S2. Morphological differences of MCF-7 sub-population during co-culture with NK cells at different E:T ratio (at 100× magnification). Inverted microscopy confirmed that NK cells reduced MCF-7, CSC, and NON-CSC cell numbers in co-cultures starting at an E:T ratio of

10:1, with widespread cell rounding and detachment. Higher E:T ratios produced no additional visible reduction compared with 10:1.
